# Structure, Spectra and Photochemistry of 2-Amino-4-Methylthiazole: FTIR Matrix Isolation and Theoretical Studies

**DOI:** 10.3390/molecules27123897

**Published:** 2022-06-17

**Authors:** Magdalena Pagacz-Kostrzewa, Daria Bumażnik, Stéphane Coussan, Magdalena Sałdyka

**Affiliations:** 1 Faculty of Chemistry, University of Wroclaw, F. Joliot-Curie 14, 50-383 Wrocław, Poland; magdalena.pagacz-kostrzewa@chem.uni.wroc.pl (M.P.-K.); darbum1@gmail.com (D.B.); 2 Aix-Marseille University, CNRS, PIIM, 13013 Marseille, France

**Keywords:** thiazole, argon matrices, DFT

## Abstract

The structure, tautomerization pathways, vibrational spectra, and photochemistry of 2-amino-4-methylthiazole (AMT) molecule were studied by matrix isolation FTIR spectroscopy and DFT calculations undertaken at the B3LYP/6-311++G(3df,3pd) level of theory. The most stable tautomer with the five-membered ring stabilized by two double C=C and C=N bonds, was detected in argon matrices after deposition. When the AMT/Ar matrices were exposed to 265 nm selective irradiation, three main photoproducts, N-(1-sulfanylprop-1-en-2-yl)carbodiimide (fp1), N-(1-thioxopropan-2-yl)carbodiimide (fp2) and N-(2-methylthiiran-2-yl)carbodiimide (fp3), were photoproduced by a cleavage of the CS–CN bond together with hydrogen atom migration. The minor photoreaction caused by the cleavage of the CS–CC bond and followed by hydrogen migration formed 2-methyl-1H-azirene-1-carbimidothioic acid (fp15). We have also found that cleavage of the CS–CN bond followed by disruption of the N–C bond produced cyanamide (fp11) and the C(CH3)=CH–S biradical that transformed into 2-methylthiirene (fp12) and further photoreactions produced 1-propyne-1-thiole (fp13) or methylthioketene (fp14). Cleavage of the CS–CC bond followed by disruption of the N–C bond produced propyne (fp22) and the S–C(NH_2_)=N biradical that transformed into 3-aminethiazirene (fp23); further photoreactions produced N-sulfanylcarbodiimide (fp25). As a result of these transformations, several molecular complexes were identified as photoproducts besides new molecules in the AMT photolysis process.

## 1. Introduction 

Thiazoles are basic heterocyclic compounds with five-membered ring including one nitrogen and one sulfur atom. Many compounds containing the thiazole system in the molecule exhibit a broad spectrum of pharmacological activity. Sulfathiazole, Ritonavir, Abafungin, Tiazofurin, or Bleomycin are often used antimicrobial, antiretroviral, antifungal, antineoplastic or antitumor drugs with the thiazole ring in the structure, respectively [1,2,3]. An important naturally occurring compound containing a thiazole ring is thiamine (i.e., vitamin B1) consisting of thiazole and pyrimidine rings, connected by a methylene group. Thiamine is an important cofactor in the enzymes of glycolysis, the Krebs cycle and the pentose phosphate pathway. It also takes part in the biosynthesis of neurotransmitters—acetylcholine and gamma-aminobutyric acid [4,5]. The latest review papers on the biological and pharmaceutical properties of 2-aminothiazoles concern both the synthesis and anti-cancer properties of these compounds [6,7,8]. Another interesting aspect in the research on thiazoles is their role in prebiotic chemistry on the early Earth [9,10]. Recently, 2-aminoazoles have been considered as intermediates in nucleotide synthesis or nucleotide activating or/and selective agents in prebiotic chemistry [11]. 2-aminothiazole selectively crystallizes the needed sugars in the presence of mixtures of aldoses and ketoses playing the role of chaperone for ribonucleotide synthesis [12].

Photochemistry of some five-membered ring containing two hetero atoms has been investigated but the complete reaction pathways have not been established yet [13]. Flash photolysis of thiazole in the gas phase gave initially the highly excited NCS molecule which acted as a precursor to the rotationally and vibrationally excited CN radical [14]. VUV photodissociation of thiazole molecule investigated by TOF-MS and photoelectron photoion coincidence spectroscopy produced several ionic species, such as C_2_H_2_S^+^, CHS^+^, CH_2_N^+^ or S^+^ [15]. Recently, the UV induced photoreactions of thiazole isolated in low temperature argon matrices were examined [16]. The major ring-opening photoreactions caused by the cleavage of the S1–C2 bond gave *syn*-2-isocyanoethenethiol or 2-isocyanothiirane as the initial photoproducts that changed into secondary photolysis products after prolonged irradiation time. When the N3–C4 bond was broken and, on one side, was followed by the cleavage of the S1–C2 bond, the CH_2_=C=S or HC≡CSH molecules were formed, and when on the other side, the C5–S1 final cleavage took place, the HN=C=S or N≡C–SH products were identified. Other minor ring-opening photoreactions were also observed.

**Scheme 1:**
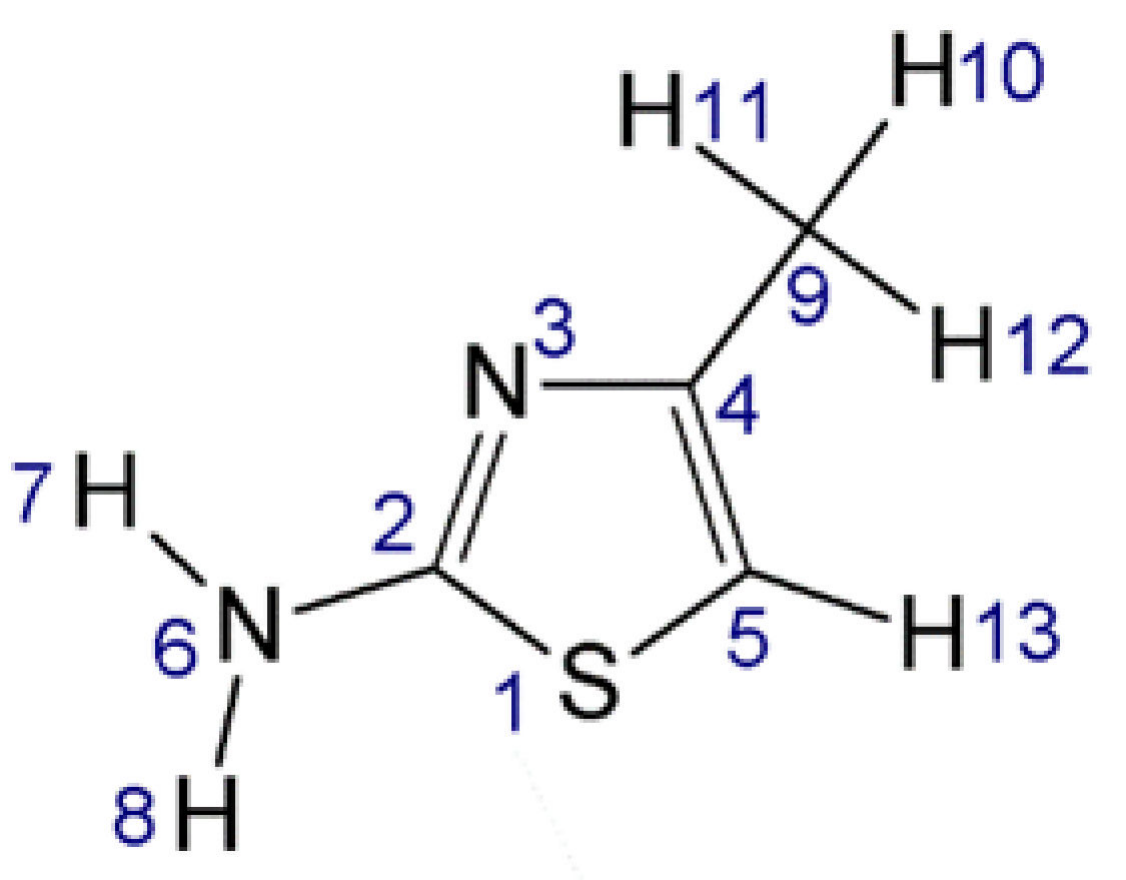
Schematic representation of the AMT molecule and the adopted atom numbering.

In the present paper we reveal a combined spectroscopic and theoretical study of the structure, vibrational spectra and photochemical behavior of 2-amino-4-methylthiazole (AMT). The structure of AMT is presented in Scheme 1.

## 2. Results and Discussion

### 2.1. Structure and Energetics

The 2-amino-4-methylthiazole molecule (AMT) can exist in several tautomeric forms, related to the migration of a proton within adjacent atoms. In the case of this heterocyclic compound there is a hydrogen atom transfer within the three-atom systems: three-carbon, imino-enamine and amidine. One may expect formation of five different types of tautomers which, following the numbering shown in Scheme 1 are denoted as AMT1, AMT2, its isomer AMT2′, AMT3, the isomeric structure AMT3′, AMT4, its isomer AMT4′ and AMT5. Full geometry optimization at the B3LYP/6-311++G(3df,3pd) level revealed eight minima. Figure S1 presents the optimized structures of the AMT minima, their energy parameters are gathered in Table 1; the selected calculated geometrical parameters of the AMT tautomers are collected in Table S1. The structures of the relevant transition states are shown in Figure S2 with the energy parameters given in Table 1. The tautomers of AMT differ by the presence of hydrogen at the N and C atoms, respectively, in both substituents and in the ring. In the case of tautomeric transformations AMT1 ↔ AMT5, AMT2 ↔ AMT4 and AMT2′ ↔ AMT4′, hydrogen transfer occurs only between carbon atoms, HC–C=C ↔ C=C–CH. In turn, in the case of AMT1 ↔ AMT2 and AMT4 ↔ AMT5 transformations, the hydrogen atom migrates within the amidine system, HN–C=N ↔ N=C–NH. On the other hand, the tautomeric transformations of AMT3’ ↔ AMT4 and AMT3 ↔ AMT4′ take place in the imino-enamine system, HN–C=C ↔ N=C–CH. The consequence of the migration of the hydrogen atom is the different position of the two double bonds within the structures of the energy minima of 2-amino-4-methylthiazole, which affects its aromaticity, ring stabilization and the stability of individual tautomers.

The obtained relative energies (ΔG_298.15_) (see Table 1) indicate that the AMT1 structure is the most stable one, which is mainly related to its aromaticity and the most effective stabilization of the five-membered ring by two double bonds C=C and C=N. AMT2, AMT2′, AMT3, AMT5, AMT3’ and AMT4, AMT4′ tautomers are characterized by much lower stability. The relative abundances of the studied AMT tautomers were calculated using equation ΔG = −RT ln K, where ΔG denotes the difference between Gibbs free energy for two tautomeric forms and K is the equilibrium constant for these species. Large energy differences induce that only AMT1 tautomer is populated to a significant extent under thermodynamic equilibrium. The populations of other tautomers would be negligibly low.

**Figure 1:**
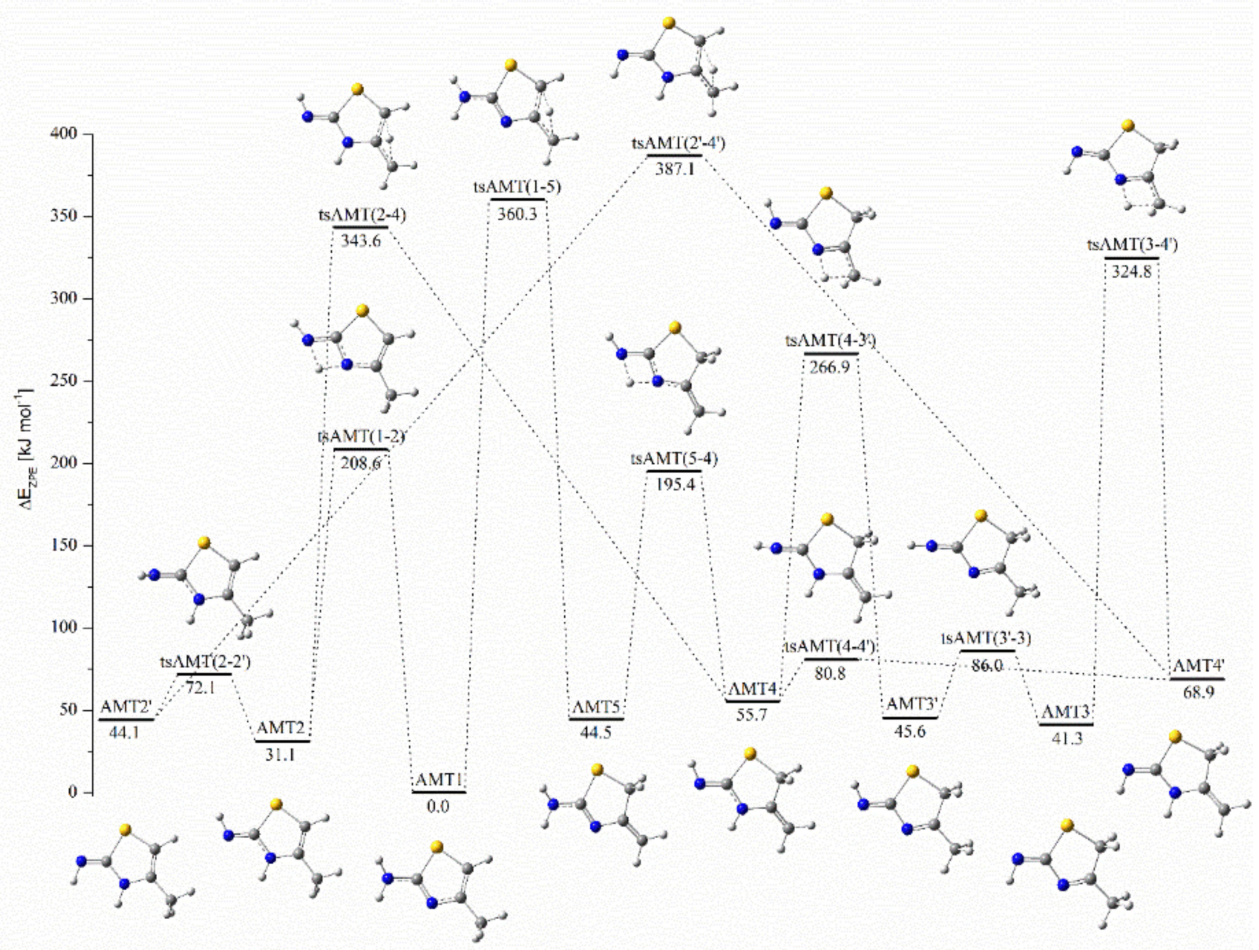
ZPE corrected potential energy diagram for AMT tautomerizations.

Figure 1 shows the ZPE corrected potential energy diagram for the tautomerization of the AMT structures. The direct transformation possible for the most stable AMT1 species include AMT1 → AMT5 tautomerization through tsAMT(1-5) proceeding by hydrogen transfer between carbon atoms of the substituent CH_3_ group and CH group of the thiazole ring with a very high energy barrier of 360.3 kJ mol^−1^. Next transformations in this reaction path AMT5 → AMT4, AMT4 → AMT3′ and AMT3 → AMT4′ include hydrogen transfer between nitrogen atoms and between nitrogen and carbon atoms via tsAMT(5-4), tsAMT(4-3′) and tsAMT(3-4′) transition states with high energy barriers of 150.9, 211.2 and 283.5 kJ mol^−1^, respectively. Hydrogen transfer processes between CH_3_ group and CH group of the thiazole ring with the very high energy barriers of 343.0 and 312.5 kJ mol^−1^ arise from AMT2′ → AMT4′and AMT2 → AMT4 reactions, respectively. The lowest energy barriers occur for transformations AMT2 → AMT2′, AMT4 → AMT4′, AMT3′ → AMT3 and are equal to 28.0, 25.1, 40.4 kJ mol^−1^, respectively. For these transformations there is no migration of hydrogen between different atoms within the molecule, but the so-called rotation on the double bond, also described in the literature as the reflection of the hydrogen atom in the isomerization reaction (One Bond Flip—OBF) [17,18]. In 2-amino-4-methylthiazole, as a result of this transformation, the position of the hydrogen atom changes only in relation to the adjacent nitrogen atom, which requires much less energy than in the case of other transformations. The most stable AMT1 may also undergo a tautomerization through tsAMT(1-2) to form AMT2 by the direct hydrogen transfer between two N atoms. The calculated energy barrier for this reaction is very high and equals 208.6 kJ mol^−1^.

### 2.2. Matrix Isolation Spectra of AMT

**Figure 2:**
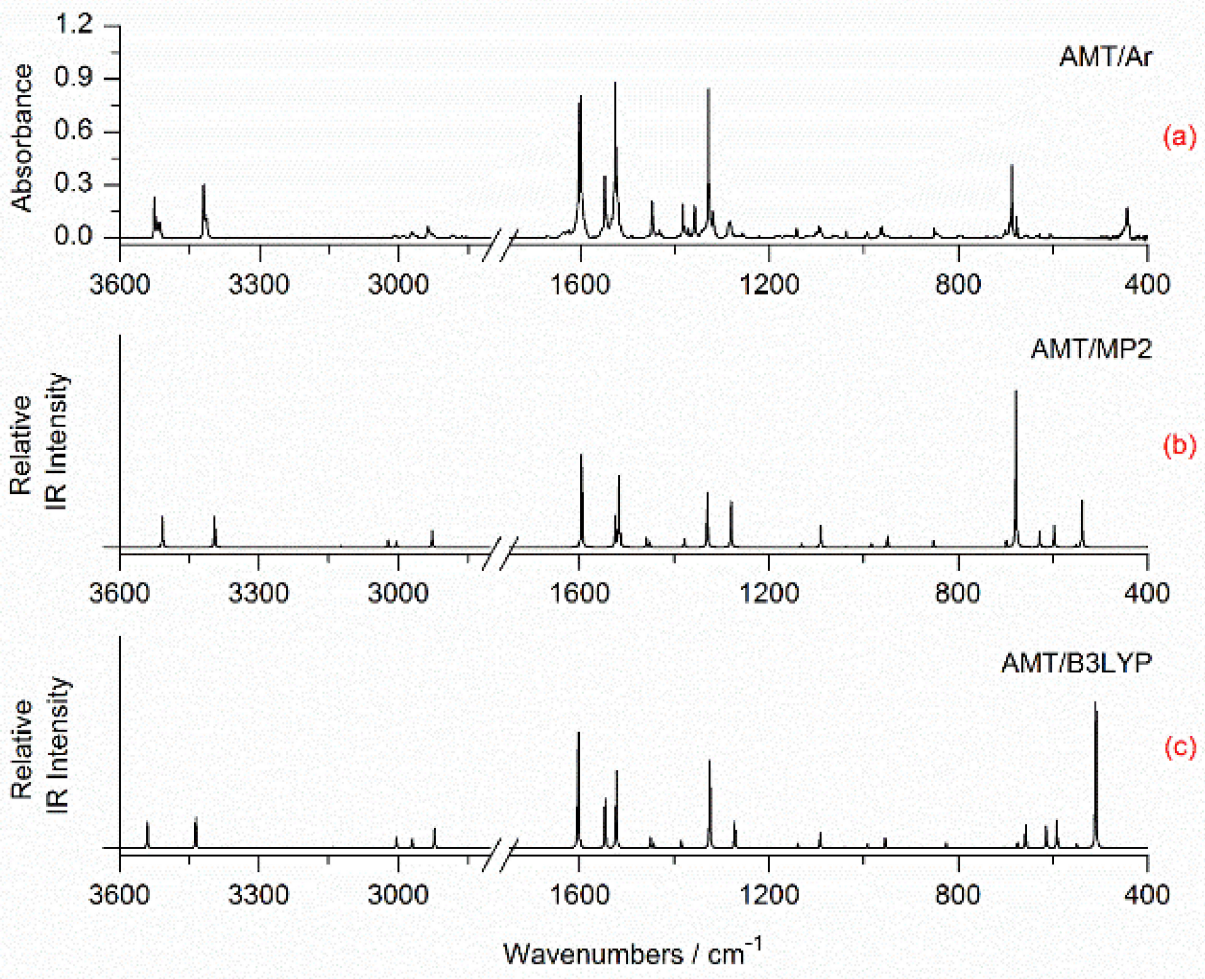
(**a**) Experimental spectrum of AMT isolated in an argon matrix deposited at 15 K and measured at 10 K; (**b**) spectrum of AMT1 tautomer calculated by the MP2/aug-cc-pVTZ method; (**c**) spectrum of AMT1 tautomer calculated by the B3LYP/6-311++G(3df,3pd) method.

The estimated relative abundance of AMT tautomers in the gas phase based on the calculated values of Gibbs free energy is presented in Table 1. According to these results only the most stable AMT1 structure is expected to be present in low temperature matrices directly after deposition. Figure 2 shows the FTIR spectrum of AMT/Ar matrix deposited at 15 K (10 K for measurement) compared to the simulated theoretical spectrum of the AMT1 tautomer. Calculated spectra of all tautomers are presented in Figure S3 for comparison. As can be seen, a satisfying reproduction of the band positions of the experimental data was obtained for the B3LYP method of calculation, therefore it was used for further calculations. In the case of the MP2 method, the values of the calculated wavenumbers differed to a greater extent from the corresponding experimental wavenumbers. However, one can see that the spectrum simulated by the MP2 method better approximates the intensity of the bands in the range below 700 cm^−1^. Most likely, the distribution of potential energy between these vibrations differs between methods. Moreover, if there are different contributions to different internal coordinates in a given vibration, this translates into changes in the dipole moment, and thus in the vibration intensity. The positions and intensities of the bands observed in the spectra of AMT/Ar matrices together with the wavenumbers predicted by the calculations for AMT1 tautomer are listed in Table 2.

### 2.3. Photolysis

**Scheme 2:**
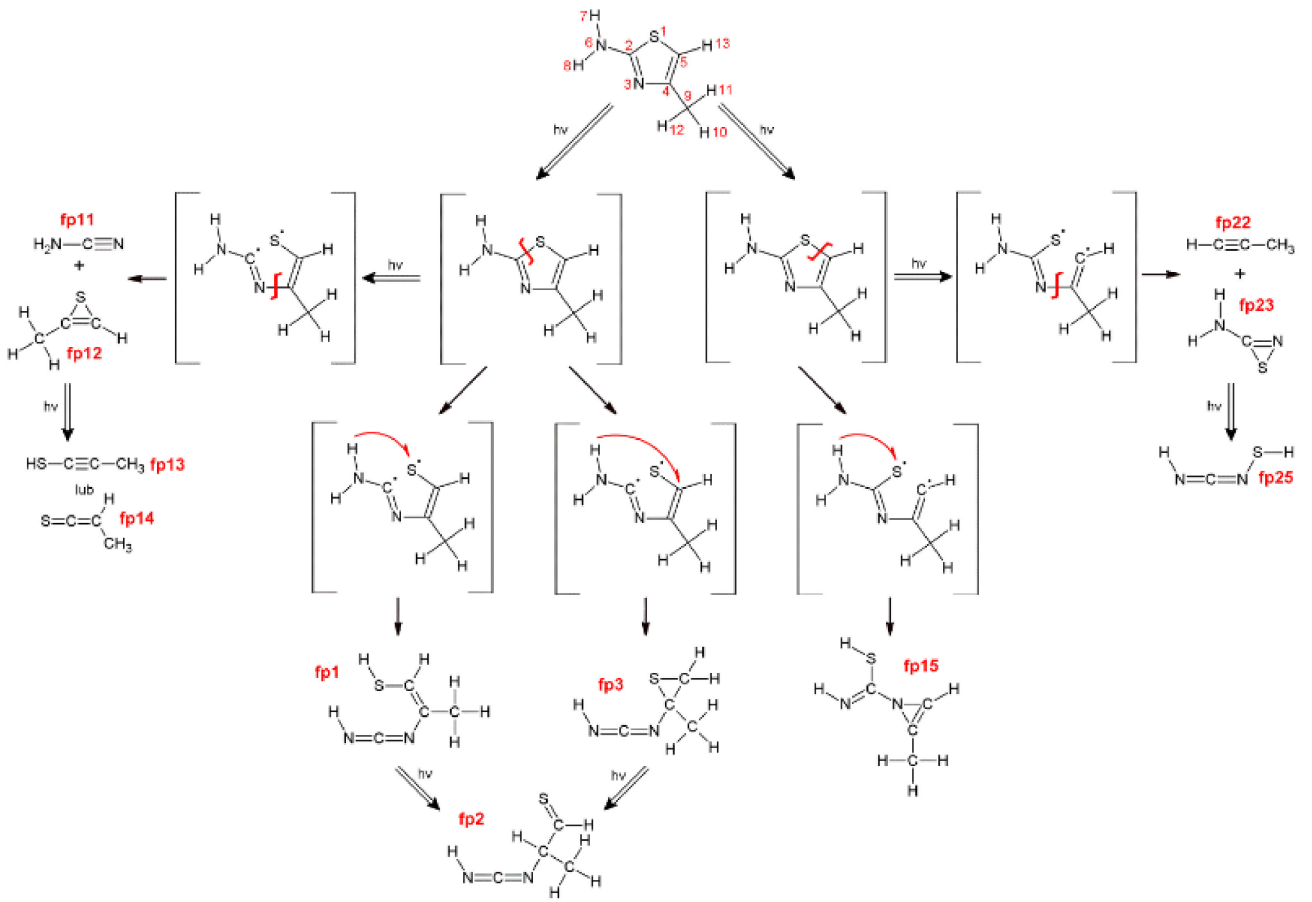
The identified photoreaction pathways of AMT.

The AMT molecules isolated in argon matrices have been irradiated using UV photons delivered by an OPO type tunable laser. The irradiations started at a radiation wavelength of 300 nm and proceeded with gradual decrease of the output wavelength. After each irradiation, an infrared spectrum of the matrix was taken. The observation of the photolysis began at 275 nm and led to the consumption of the AMT1 tautomer. However, the rate of the reaction was relatively low. Further irradiation at 265 nm led to a pronounced decrease of the intensity of the AMT1 bands and concomitantly to the growth of new bands with simultaneous appearance and increase of new bands in the spectra. The behavior of the most representative bands during photolysis is shown in Figure S4. These experiments allowed for interesting photo-transformations to be detected. Literature reports on photochemical rearrangements of thiazole, thiazole derivatives and five-membered ring heterocycles in general [13,16,19,20,21,22], allowed to propose the photoreaction pathways of 2-amino-4-methylthiazole. All proposed paths of photolysis of AMT are presented in Figure S5 and Figure S6. The structures of all photoproducts, named fp1-fp41, were optimized using the B3LYP/6-311++G(3df,3pd) method; their harmonic wavenumbers were also calculated and gathered in Table S2. The analysis of the most intense and characteristic bands for these molecules and comparing their position with new product bands appearing in the experimental spectrum allowed for rejection of the following structures from further considerations: fp4-fp10, fp16-fp21 and fp26-fp41. The photoproduct bands of AMT were identified and assigned to fp1, fp2, fp3, fp11-fp15 and fp22-fp25 structures. Scheme 2 presents the identified photoreaction pathways of AMT.

**Ring-opening reactions by cleavage of the S1–C2 bond**

**Figure 3:**
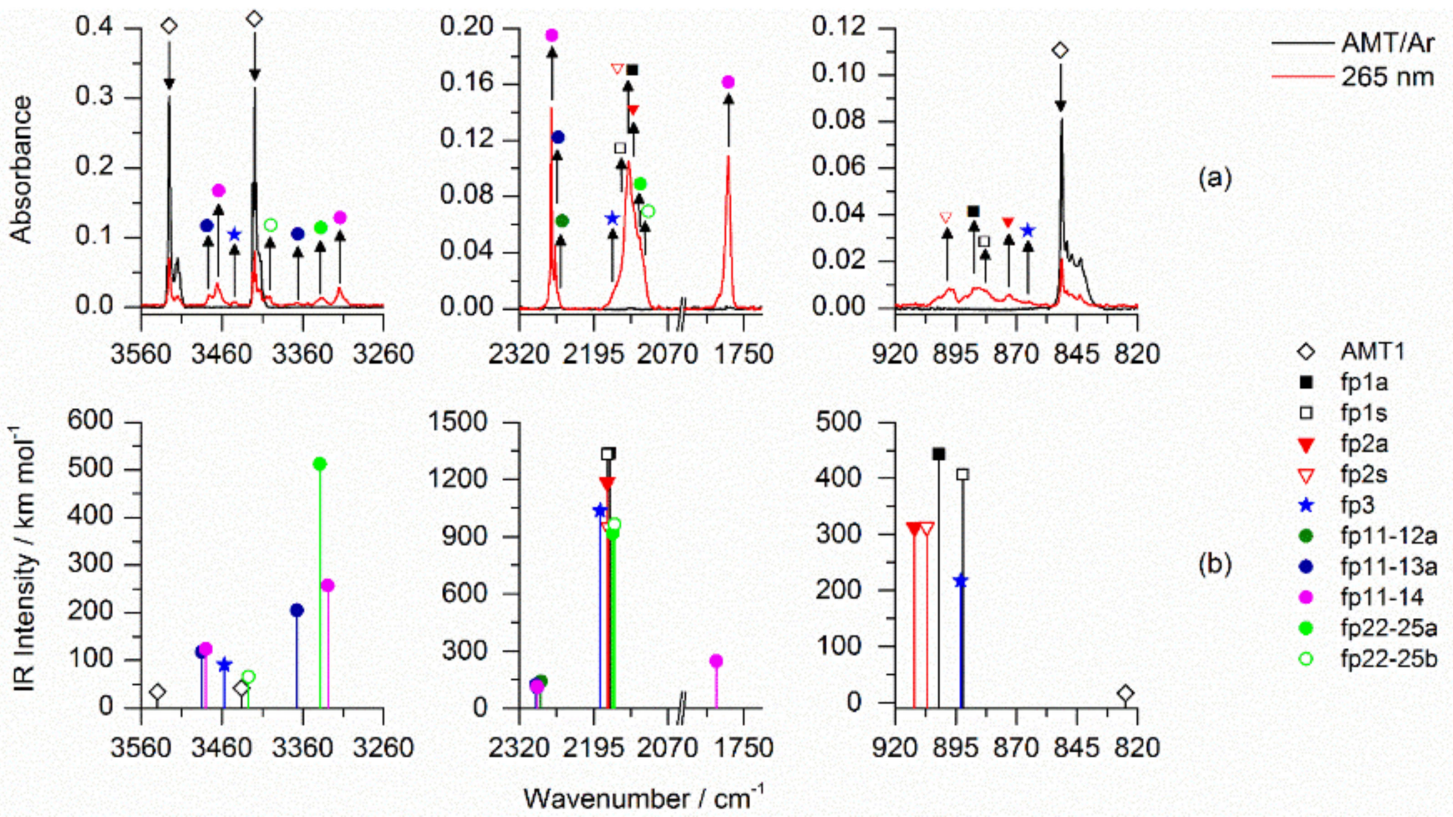
Selected regions of the AMT/Ar spectra: (**a**) after deposition at 15 K/10 K (black traces) and after 300 min irradiation at 265 nm (red traces), (**b**) B3LYP/6-311++G(3df,3pd) stick spectra of the identified photoproducts.

**Figure 4:**
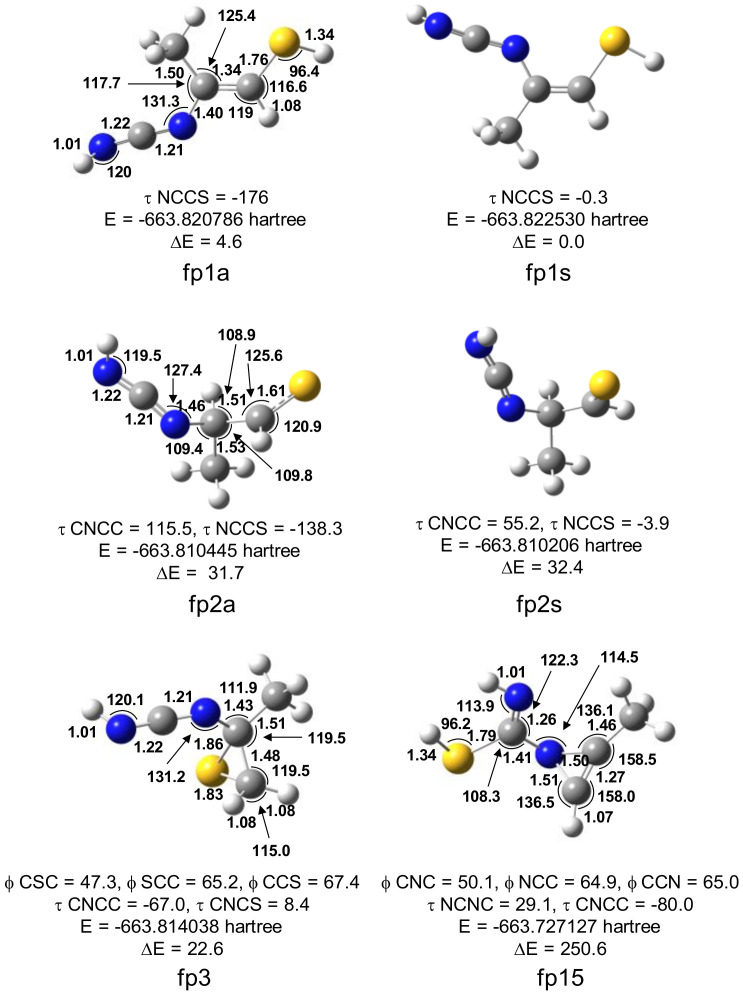
Optimized structures, energies (in hartree) and relative energies ΔE (in kJ mol^−1^) of the photoproduct molecules. The numbers in figures represent bond lengths (in Ångstroms) and angles (in degrees). The defined dihedral angles are in degrees.

*Identification of new carbodiimides containing sulfur atom.* Figure 3 and Figure S7 present the most characteristic regions of the IR spectrum where new bands can be observed. The most prominent photoproduct absorptions appeared in the 2300–2000 cm^−1^ region. The intense, broad band at ca. 2135 cm^−1^ was identified after 5 min of irradiation at 265 nm and its intensity grew during 180 min of photolysis at 265 nm and then slightly diminished up to 300 min of during further irradiation, which could be the result of other AMT photoreactions leading to the sample decomposition in the studied matrices. The behavior of this band during photolysis is shown in Figure S8. This intense band is characteristic of structures containing the isocyano, cyano or carbodiimide group [16,21,22,23,24]. Considering the cleavage of the S1–C2 bond as the mechanism of initial photoproduct formation postulated for the thiazole molecule, similar reaction can be proposed for AMT. The experimental wavenumbers compared with calculated harmonic and anharmonic wavenumbers of the photoproduct molecules observed after photolysis of AMT/Ar matrix are gathered in Table 3. Optimized structures of the photoproduct molecules are presented in Figure 4. Anharmonic wavenumbers calculated for the identified photoproducts are collected in Table S3.

After disruption of the S1–C2 bond the hydrogen atom can migrate from N6 to S1 giving N-(1-sulfanylprop-1-en-2-yl)carbodiimide (fp1) as photoproduct (see Scheme 2). The optimization resulted in two conformers of fp1 with *E* and *Z* conformation around C=C bond (fp1a and fp1s, respectively), with the small relative energy difference ΔE_ZPE_ = 3.9 kJ mol^−1^. The comparison of the calculated spectral pattern of the fp1a and fp1s with the experimental spectrum allowed for assignment of 2134, 887.5 and 644 cm^−1^ bands to fp1a and 2142, 885 cm^−1^ band to fp1s products, respectively. When the hydrogen atom migrates from N6 to C5 after the cleavage of the S1–C2 bond the N-(2-methylthiiran-2-yl)carbodiimide molecule (fp3) can be formed. A careful analysis of the spectrum revealed bands of fp3 photoproduct at 3443.5, 2160, 1373.5, 1375.5 and 865.0 cm^−1^. Additionally, several absorptions due to N-(1-thioxopropan-2-yl)carbodiimide (fp2) can be also observed. On the basis of calculations broad absorption at 2130 cm^−1^ and 1080.5, 1000.0 and 873.0 cm^−1^ bands were attributed to fp2a photoproduct, while 2137, 1263.0, 1057.5, 998.5, 898.0 and 644 cm^−1^ bands were assigned to the fp2s structure. Both conformers of fp2 differ in position of the H–C=S group in relation to the carbodiimide group, and thus they vary in the values of the NCCS torsion angle (−138.30 for fp2a, −3.90 for fp2s), which gives a very small electronic energy difference ΔE_ZPE_ = 0.2 kJ mol^−1^ for these structures.

*Formation of cyanamide complexes.* Cleavage of the S1–C2 bond followed by disruption of the N3–C4 bond (see Scheme 2) produces cyanamide molecule (fp11) and C(CH_3_)=CH-S biradical which changes into 2-methylthiirene (fp12). Further photoreactions can produce 1-propyne-1-thiole (fp13) or methylthioketene (fp14). Indeed, the spectral analysis revealed several new vibrational features present in the typical regions of cyanamide (H_2_N–C≡N) absorptions. The performed experiments allowed to distinguish three groups of bands, labeled as fp11-12, fp11-13 and fp11-14, (see Figure 3 and Figure S7) which respond in different ways to the photolysis process. The absorptions of group fp11-12 appeared after 2 min of irradiation at 265 nm as very weak bands, very slowly increasing its intensity up to 300 min of irradiation. The bands of group fp11-13 were observed after 1 min of irradiation at 275 nm, they visibly grew during irradiation at 265 nm. The absorptions of group fp11-14 appeared after 1 min of irradiation at 275 nm and grew clearly as well defined bands during irradiation at 265 nm. The absorptions attributed to group fp11-12 appeared at 3165, 2256.0, 2252.0 and 665.0 cm^−1^. The bands due to group fp11-13 were observed at 3475.0, 3365 and 2260.0 cm^−1^. The absorptions of group fp11-14 were identified at 3466.0, 3314.0, 2267.0, 1777.0 and 638.0 cm^−1^. As will be discussed below the bands due to group fp11-12 can be assigned to the H_2_N–C≡N + CH_3_–CSC–H complex, the bands of group fp11-13 can be attributed to the H_2_N–C≡N + CH_3_C≡CSH complex and the bands of group fp11-14 to the H_2_N–C≡N + S=C=C(H)CH_3_ complex.

**Figure 5:**
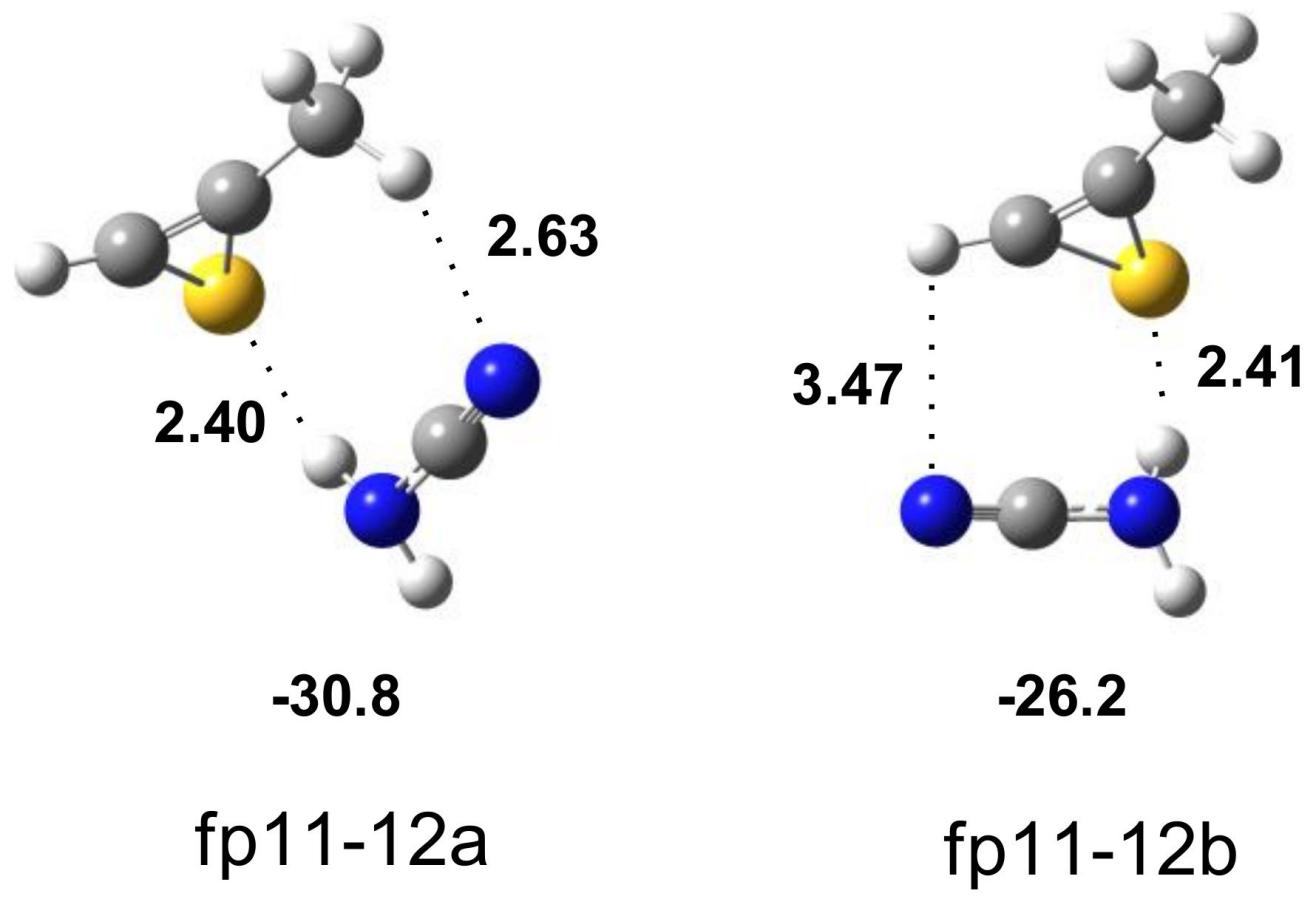
B3LYP/6-311++G(3df,3pd) optimized structures of the H_2_N–C≡N∙∙∙CH_3_-CSC–H complex. The selected bond distances (in Å) and interaction energies ΔE^CP^ (in kJ mol^−1^) are given.

*Formation of H_2_N*–*C≡N∙∙∙CH_3_-CSC-H complex*. The calculations resulted in two local minima on the potential energy surface of the cyanamide-2-methyltiirene system that correspond to the stable structures presented in Figure 5. In the more stable structure, fp11-12a (ΔE^CP^ = −30.8 kJ mol^−1^), the NH group of cyanamide serves as a proton donor toward the sulfur atom and as a proton acceptor for the CH_3_ group of 2-methyltiirene. In turn, in the fp11-12b configuration, (ΔE^CP^ = −26.2 kJ mol^−1^), similar NH∙∙∙S and CH∙∙∙N bonds are present with the CH group of 2-methyltiirene interacting with cyanamide. The full sets of harmonic vibrational wavenumbers of the optimized structures are presented in Table S2. The comparison of the experimental spectra (bands assigned to the group fp11-12) with the calculated for the two structures evidences that the complex has the structure fp11-12a. In Table 4 the theoretical wavenumber shifts, Δν_theor_ = (ν_comp_ − ν_mon_)_theor_ for both optimized structures are compared with the experimental ones, Δν_exp_ = (ν_comp_ − ν_mon_)_exp_. The experimental wavenumber for the H_2_N–C≡N molecule was taken from reference [24,25]. The formation of the structure fp11-12a in the matrix is reflected by the appearance of the bands due to perturbed ν_s_NH_2_, νC≡N and ωNH_2_ modes of cyanamide. The 3165 and 2256.0, 2252.0 cm^−1^ absorptions assigned to the perturbed NH_2_ symmetric stretching and C≡N stretching vibrations are 235 and ca. 10 cm^−1^ red shifted and the 665.0 cm^−1^ band attributed to the NH_2_ wagging mode is 251 cm^−1^ blue shifted with the corresponding modes of the H_2_N–C≡N monomer, respectively. As one can see in Table 4 the observed pattern of the band shifts is better reflected by the shifts of the more stable complex of structure fp11-12a, particularly the observed shift of the ωNH_2_ band (Δν_obs_ = +251 cm^−1^) shows better agreement with the one calculated for this structure (Δν_calc_ = +206 cm^−1^). Unfortunately, none of the 2-methylthiirene band was identified in the spectra but it seems to be justified by the low intensities of the absorptions of this molecule: the calculated intensity of the most intense band of CH_3_-CSC-H does not exceed 40 km mol^−1^ for both the monomer and the complexes. So, most probably absorptions of 2-methyltiirene are overlapped by other bands.

**Figure 6:**
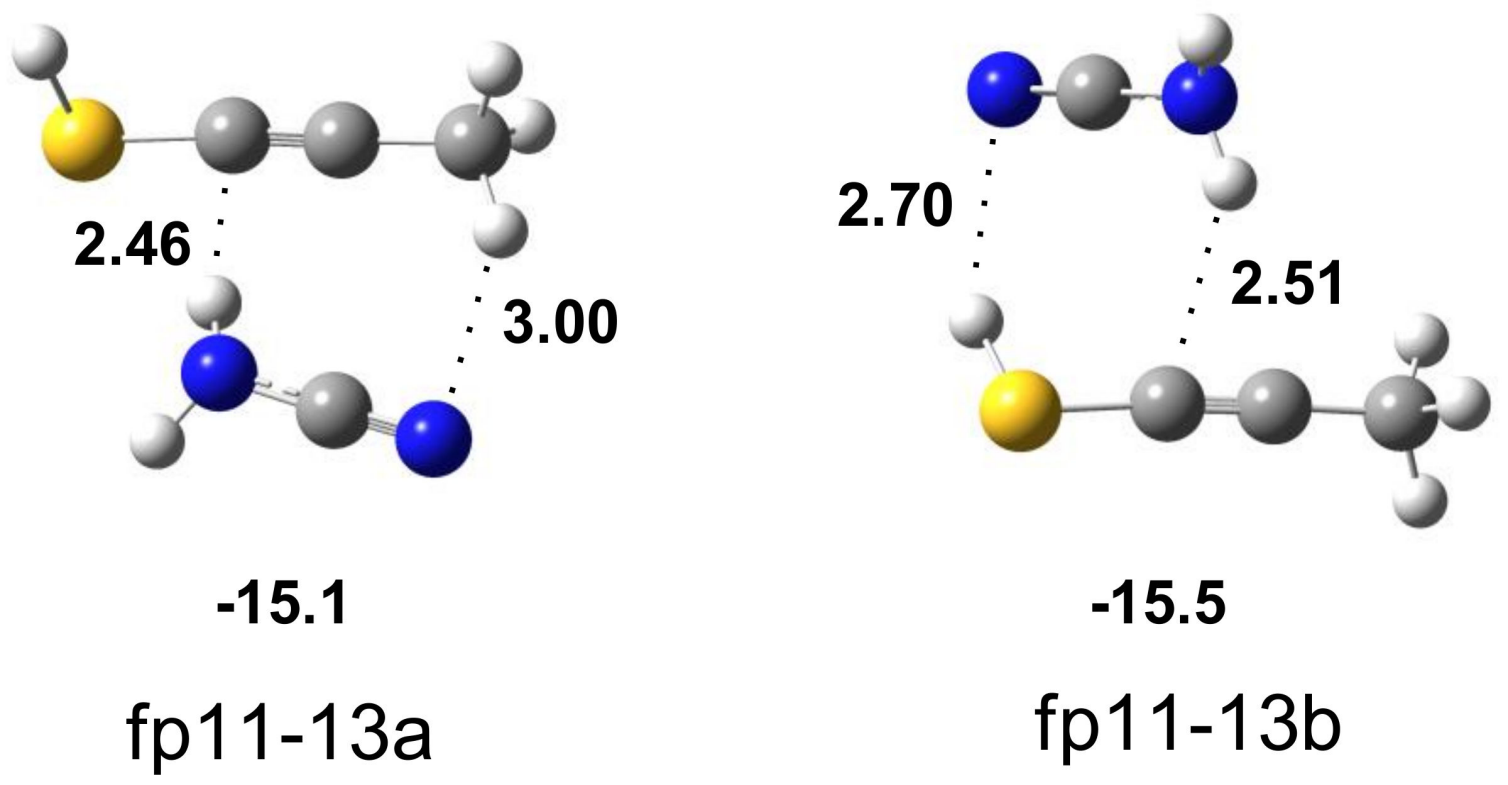
B3LYP/6-311++G(3df,3pd) optimized structures of the H_2_N–C≡N∙∙∙CH_3_C≡CSH complex. The selected bond distances (in Å) and interaction energies ΔE^CP^ (in kJ mol^−1^) are given.

*Formation of the H_2_N–C≡N∙∙∙CH_3_C≡CSH complex.* Figure 6 presents two structures corresponding to the stationary points calculated for the cyanamide complex with 1-propyne-1-thiol. In both structures the NH group of cyanamide acts as a weak proton donor towards the C≡C bond. In structure fp11-13a (ΔE^CP^ = −15.1 kJ mol^−1^) H_2_N–C≡N plays also a role of proton acceptor of the CH_3_ group of CH_3_CCSH and in slightly more stable structure fp11-13b (ΔE^CP^ = −15.5 kJ mol^−1^) 1-propyne-1-thiol forms the SH∙∙∙N bond with cyanamide. The full sets of vibrational wavenumbers of the optimized structures are presented in Table S2. In Table 5 the theoretical wavenumber shifts for both optimized structures are compared with the experimental ones. The comparison of the experimental spectra (bands assigned to the group fp11-13) with the calculated for the two structures indicates that the complex trapped in the matrix has the structure fp11-13a. Three distinct bands due to the H_2_N–C≡N∙∙∙CH_3_C≡CSH complex appeared in the spectral regions of cyanamide: at 3475.0 cm^−1^, (Δν_exp_ = −11 cm^−1^), in the ν_as_NH_2_ region; at 3365 cm^−1^, (Δν_exp_ = −35 cm^−1^), in the vicinity of ν_s_NH_2_ and at 2260.0 cm^−1^, (Δν_exp_ = −4 cm^−1^) in the region of the νC≡N vibration, respectively. The observed wavenumber shifts match reasonably well the values calculated for the fp11-13a structure: Δν_calc_ = −22, −54 and −8 cm^−1^, respectively. In spite of the very careful spectral analysis, no bands attributed to the perturbed 1-propyne-1-thiole molecule were identified, which may be caused by the overlapping of its bands with other absorptions as the calculations predict low intensity bands for this compound (see Table S2).

**Figure 7:**
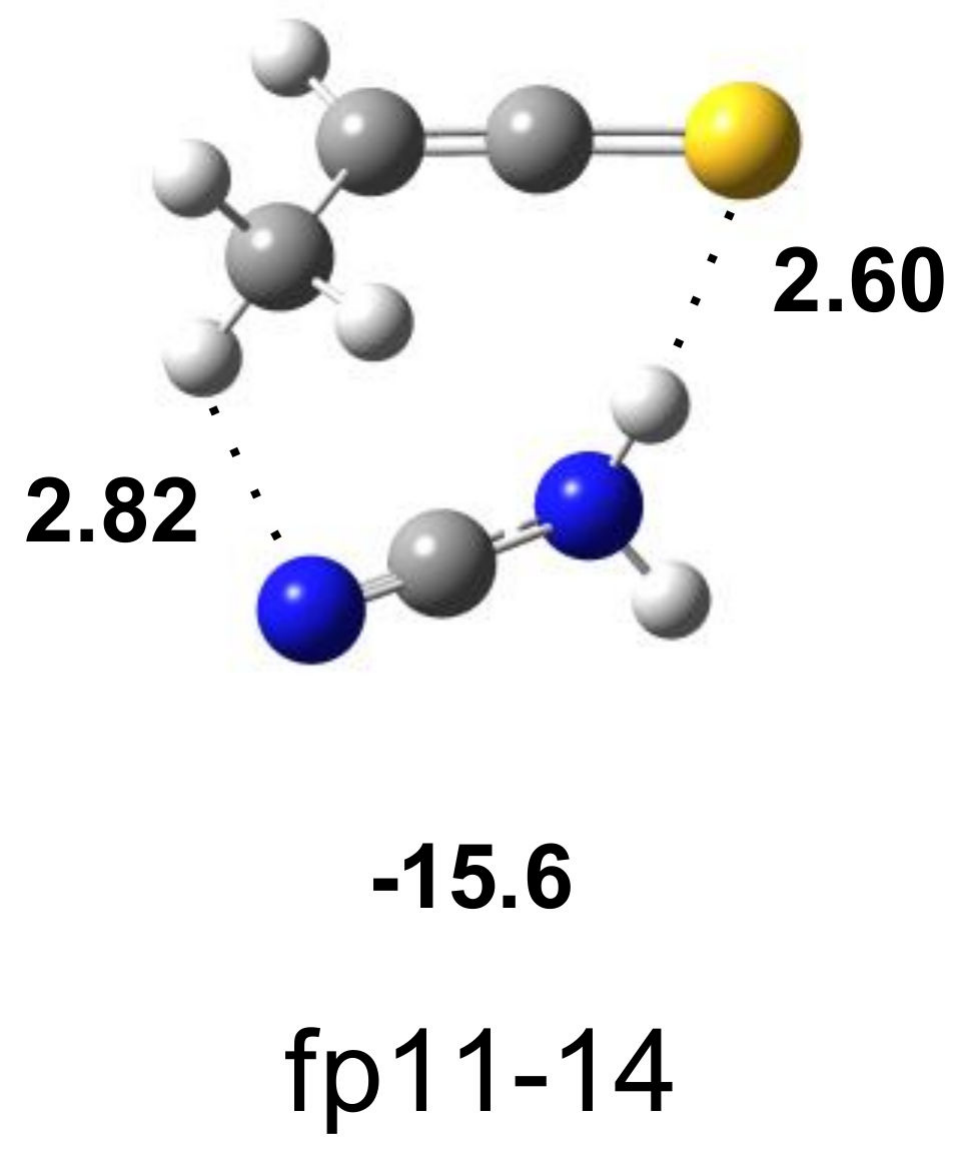
B3LYP/6-311++G(3df,3pd) optimized structure of the H_2_N–C≡N∙∙∙S=C=C(H)CH_3_ complex. The selected bond distances (in Å) and interaction energies ΔE^CP^ (in kJ mol^−1^) are given.

*Formation of the H_2_N–C≡N∙∙∙S=C=C(H)CH_3_ complex.* The calculations resulted in one global minimum on the potential energy surface of the cyanamide complex with methylthioketene (ΔE^CP^ = −15.6 kJ mol^−1^) presented in Figure 7. In this structure the NH∙∙∙S bond is formed and a weak interaction of the CH_3_ group of S=C=C(H)CH_3_ with the nitrogen atom of CN group of H_2_N–C≡N is also present. In Table 6 the theoretical wavenumber shifts for the fp11-14 structure are compared with the experimental ones. The cyanamide and methylthioketene monomer wavenumbers were taken from references [24,25,26]. The full set of harmonic vibrational wavenumbers of the optimized structure is presented in Table S2. In the region of the νC≡N vibration a distinct band grows after matrix irradiation (at 2267.0 cm^−1^) which suggests formation of one structure. The suggestion is supported by appearance of one intense band for the ν_as_C=C=S vibration of S=C=C(H)CH_3_ (1777.0 cm^−1^) and for the ν_as_NH_2_ and ν_s_NH_2_ vibrations of H_2_N–C≡N (3466.0 and 3314.0 cm^−1^, respectively). The 638.0 cm^−1^ band due to the perturbed γCH vibration of methylthioketene was also observed. As one can see in Table 6 the experimental shifts are in accord with the theoretical ones both for the cyanamide and for methylthioketene molecules.

**Ring-opening reactions by cleavage of the S1–C5 bond**

*Identification of sulfur compound with the azireno group.* After cleavage of the S1–C5 bond the hydrogen atom can migrate from N6 to S1 giving 2-methyl-1H-azirene-1-carbimidothioic acid (fp15) as photoproduct (see Scheme 2). As one can see in Figure S7 only three absorptions were observed for fp15, they were identified at 1618.0, 1250.0 and 1103.0 cm^−1^. These bands appeared after 2 min of irradiation at 270 nm and their intensity grew during 300 min of photolysis at 265 nm. The performed optimization resulted in four stable structures of fp15 with different conformations of both SH and NH groups around the C=N double bond. The relative energy differences (ΔE_ZPE_) for these structures are: 0.0, 2.4, 3.2 and 7.2 kJ mol^−1^ for the fp15*anti(SH)*/*syn(NH)*, fp15*syn*/*anti*, fp15*syn*/*syn* and fp15*anti*/*anti* forms, respectively. The comparison of the experimental bands with the absorptions calculated for the conformers of fp15 suggests that the *syn*/*syn* form of 2-methyl-1H-azirene-1-carbimidothioic acid is present in the matrix (see Table 3).

*Formation of propyne complexes.* Cleavage of the S1–C5 bond followed by disruption of the N3–C4 bond (see Scheme 2) produces the propyne molecule (fp22) and S-C(NH_2_)=N biradical which changes into 3-aminethiazirene (fp23). Further photoreactions can produce thiaziridin-3-imine (fp24) or N-sulfanylcarbodiimide (fp25). Based on the spectra calculated for the possible propyne complexes, the complex between propyne and N-sulfanylcarbodiimide (fp22-25) was identified in the AMT/Ar matrix after photolysis since the spectral analysis revealed several new vibrational features characteristic for the carbodiimide modes. However, the formation of the 3-aminethiazirene molecule (in fp22-23) in the matrix cannot be completely ruled out due to the presence of a broad shoulder at 1792 cm^−1^ on an intense 1777 cm^−1^ band observed for the fp11-14 complex (see the discussion above). The 1792 cm^−1^ absorption might be assigned to the strongest band of the ν_as_NCN vibration in the fp22-23 complex for which the calculated intensity exceeds 200 km mol^−1^.

**Figure 8:**
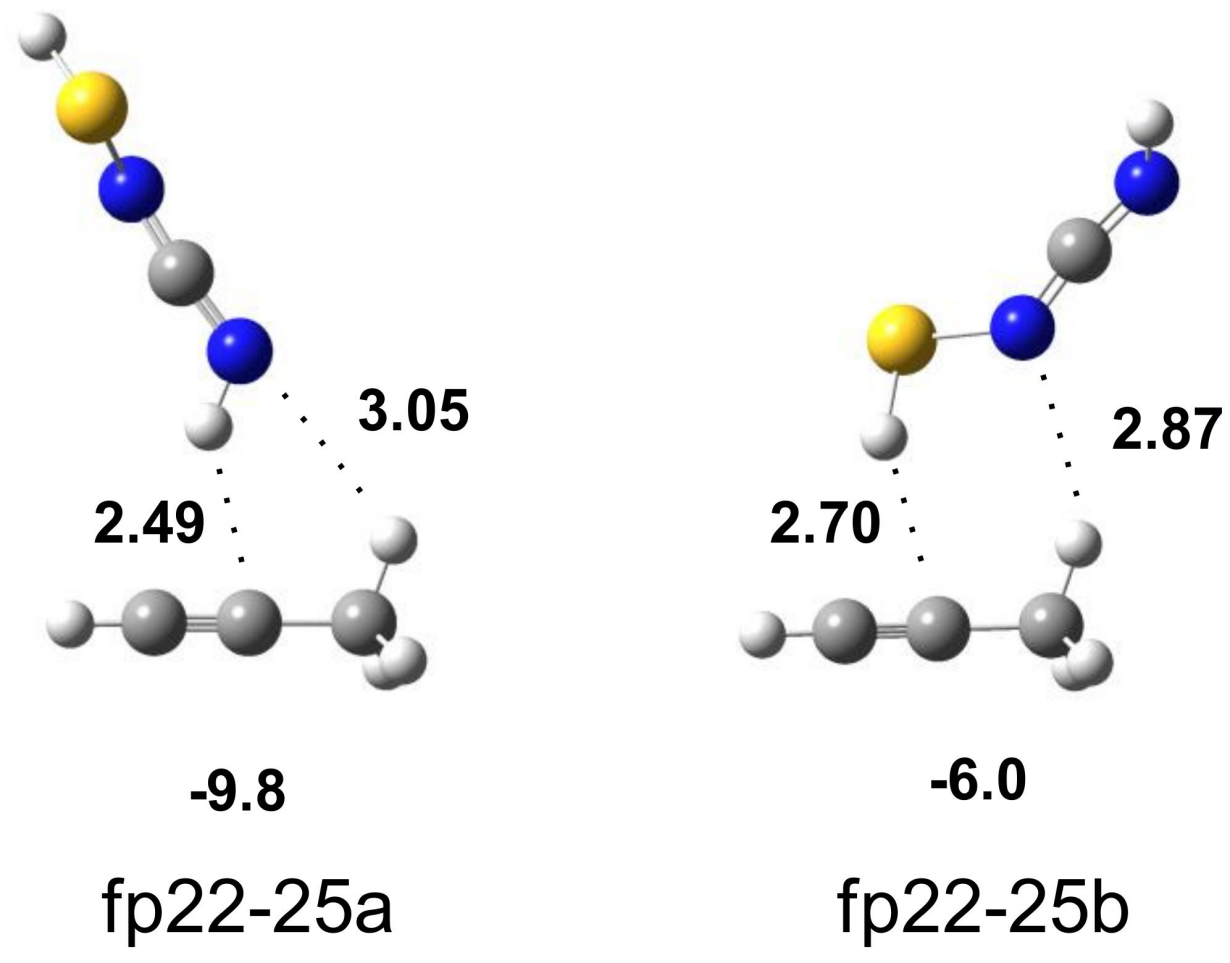
B3LYP/6-311++G(3df,3pd) optimized structure of the H_3_C–C≡CH∙∙∙HN=C=N–SH complex. The selected bond distances (in Å) and interaction energies ΔE^CP^ (in kJ mol^−1^) are given.

*Formation of the H_3_C–C≡CH∙∙∙HN=C=N–SH complexes.* The calculations resulted in two local minima on the potential energy surface of the propyne—N-sulfanylcarbodiimide system that correspond to the stable structures presented in Figure 8. In the more stable structure, fp22-25a (ΔE^CP^ = −9.8 kJ mol^−1^), the NH group of N-sulfanylcarbodiimide serves as a proton donor toward the C≡C triple bond and as a proton acceptor for the CH_3_ group of propyne. In turn, in the fp22-25b configuration, (ΔE^CP^ = −6.0 kJ mol^−1^), the SH group plays the role of proton donor toward the C≡C triple bond and the nitrogen atom is the proton acceptor of the CH_3_ group of propyne. The full sets of harmonic vibrational wavenumbers of the optimized structures are presented in Table S2. The propyne monomer wavenumbers were taken from references [27,28]. The experimental spectra of HN=C=N–SH have not been reported so far and the Δν_exp_ values for this molecule could not be estimated. So, in this case the theoretical anharmonic wavenumbers of HN=C=N–SH were taken to Δν_exp_ calculations instead of the missing monomer experimental wavenumbers. The anharmonic wavenumbers calculated for HN=C=N–SH are presented in Table S3. In Table 7 the theoretical wavenumber shifts for both optimized structures are compared with the experimental ones. As one can see, a reasonable agreement can be noticed between the observed shifts and calculated for the structure fp22-25a. Some inconsistency in these values may be due to the lack of experimental wavenumbers for N-sulfanylcarbodiimide. The anharmonic wavenumber of the ν_as_NCN vibration in the monomer (2163 cm^−1^) is much higher than the identified wavenumber of the perturbed ν_as_NCN vibration in the complex (2117.5 cm^−1^). Most likely the anharmonicity of this vibration is greater than the calculations predict, which may be due to the coupling of the stretching vibrations of the NCN group and the NH group present in the immediate vicinity. A similar situation occurs for fp1 or fp2 photoproducts, for which the ν_as_NCN vibration bands were observed at ca. 2135 cm^−1^, and the calculations predicted the anharmonic wavenumbers between 2182 and 2158 cm^−1^ for this mode. The 898 cm^−1^ band was only tentatively assigned for the complex since this wavenumber coincides with the δNH bands in other carbodiimide molecules present in the matrix. The 635.0 cm^−1^ band due to the perturbed δCH/γCH vibration of propyne was also observed. As can be seen in Figure 3, there is some evidence that the complex of configuration fp22-25b is also present in the matrix: the 3400.0 and 2112.0 cm^−1^ bands can be tentatively assigned to the perturbed νNH and ν_as_NCN vibrations in this complex, respectively.

## 3. Materials and Methods

2-amino-4-methylthiazole was commercially available (98%, Sigma-Aldrich, Merck, KGaA, Darmstadt, Germany). The AMT/Ar matrices were prepared by passing of high purity argon through the glass U-tube with AMT kept at room temperature and situated outside the cryostat chamber. The AMT/Ar gaseous mixtures were deposited onto a cold CsI window kept at a temperature 15 K/10 K (deposition/measurement) by means of a closed cycle helium refrigerator (ARS-2HW, APD-Cryogenics). Infrared spectra were recorded between 4000–400 cm^−1^ in a transmission mode with a resolution of 0.5 cm^−1^ by means of a Nicolet iS50 FTIR spectrometer equipped with a liquid N_2_ cooled MCT detector.

After the infrared spectra of the initially deposited matrices were recorded the samples were irradiated with the tunable UV radiation provided by the frequency doubled signal beam of a pulsed (7 ns) optical parametric oscillator Vibrant 355 (Opotek Inc., Carlsbad, CA, USA) (repetition rate 10 Hz, average pulse energies ~7.0 mJ (325 nm) and ~2.5 mJ (250 nm)) pumped with a pulsed Nd:YAG laser (Quantel).

All calculations were performed with the Gaussian 09 suit of programs [29]. Structures of the minima and transition states were optimized at the B3LYP/6-311++G(3df,3pd) level of theory. On the basis of the force constant matrixes calculated at the same level, harmonic frequencies were evaluated as well as zero-point vibrational energy (ZPE) corrections and the corresponding thermodynamic functions. The transition states linking the tautomers were localized on the PES and verified by the presence of one imaginary vibrational frequency. The corresponding minima were connected to each TS by following intrinsic reaction coordinate (IRC). For the most stable tautomer AMT1 the structure and vibrational spectrum were also calculated at the MP2/aug-cc-pVTZ level. In order to account for anharmonic effects, the calculated vibrational frequencies were scaled by a factor of 1.0/1.0 (for wavenumbers in the range from 400 to 750 cm^–1^), 0.978/0.973 (for wavenumbers in the range from 750 to 2500 cm^−1^) or 0.964/0.953 (for wavenumbers above 2500 cm^–1^) for B3LYP/MP2 calculations, respectively. The vibrational spectra were simulated using SYNSPEC program [30]. Anharmonic wavenumbers were calculated for the selected monomeric species at the B3LYP/6-311++G(3df,3pd) level of theory. The structures of the complexes were also optimized at the B3LYP level and their binding energies were corrected by the Boys-Bernardi full counterpoise procedure (CP) [31]. According to the reviewer’s suggestion, we re-computed the electronic energy values at the B3LYP-D3(BJ) level to get ΔE^CP^ (in kJ mol^−1^) for the structures of complexes obtained after optimization at B3LYP/6-311++G(3df,3pd). The new energy values are presented in Table S4, Supplementary Materials. As can be seen, introduction of D3 dispersion correction to calculations reduces the binding energy in complexes, but overall the order of the structures in terms of energetics does not change.

## 4. Conclusions

Matrix isolation FT-IR spectroscopy, supported by B3LYP/6-311++G(3df,3pd) calculations, allowed for the first time to observe and characterize spectroscopically the AMT1 tautomer of 2-amino-4-methylthiazole which owes its stability to aromaticity and effective stabilization of the five-membered ring by C=C and C=N double bonds.

The narrow band UV irradiation technique was applied to study the photochemistry of 2-amino-4-methylthiazole. There are two main mechanisms that initiate photochemical reactions. The major ring-opening photoreactions caused by the cleavage of the S1–C2 bond lead to the formation of C(NH_2_)=N–C(CH_3_)=CH–S biradical. The hydrogen atom of the NH_2_ group of the radical migrates to S1 to form N-(1-sulfanylprop-1-en-2-yl)carbodiimide (fp1a, fp1s) or to C5 to produce N-(2-methylthiiran-2-yl)carbodiimide (fp3); subsequent photochemical reactions may transform both fp1, fp3 photoproducts into N-(1-thioxopropan-2-yl)carbodiimide (fp2a, fp2s) as shown in the Scheme 2. Cleavage of the S1–C2 bond followed by disruption of the N3–C4 bond produces the cyanamide molecule and the ˙C(CH_3_)=CH–S˙ biradical that transforms into 2-methylthiirene (fp12); further photoreactions can produce 1-propyne-1-thiole (fp13) or methylthioketene (fp14). The minor ring-opening photoreaction is caused by the cleavage of the S1–C5 bond upon UV irradiation. The hydrogen atom migration from N6 to S1 in S–C(NH_2_)=N–C(CH_3_)=CH forms 2-methyl-1H-azirene-1-carbimidothioic acid (fp15) as photoproduct. Cleavage of the S1–C5 bond followed by disruption of the N3–C4 bond produces the propyne molecule and S–C(NH_2_)=N biradical that transforms into 3-aminethiazirene (fp23). Further photoreactions produce N-sulfanylcarbodiimide (fp25). As the above studies showed, apart from new photoproduct molecules obtained in the AMT photolysis process, several molecular complexes were also identified as photoproducts. As the two co-products of photodissociation channel of a molecule are trapped in one cage they may form a complex. The fact why most of the co-products formed in the same matrix cage create complexes but some stay as monomers was discussed widely in our earlier papers [32,33,34,35]. It is worth mentioning that cyanamide [36] and propyne [37] molecules have been identified in space, so their molecular complexes presented in this paper may have potential significance for astrophysics, astrochemistry or even astrobiology.

## Figures and Tables

**Table 1:** Relative energies (Δ*E*), relative zero-point corrected energies (Δ*E*_ZPE_), relative Gibbs free energies (Δ*G*) (in kJ mol^−1^) and abundances at 298 K for 2-amino-4-methylthiazole tautomers and transition states calculated at the B3LYP/6-311++G(3df,3pd) level.

**Minima**	**Δ*E***	**Δ*E*_ZPE_**	**Δ*G*_298.15_**	**%_298_**
AMT1	0.0	0.0	0.0	100
AMT2	31.3	31.1	30.8	0
AMT2′	45.3	44.1	43.8	0
AMT3	42.5	41.3	40.3	0
AMT3′	46.1	45.6	44.5	0
AMT4	54.5	55.7	54.6	0
AMT4′	68.5	68.9	68.0	0
AMT5	44.4	44.5	42.6	0
**TS**	**Δ*E***	**Δ*E*_ZPE_**		**ν_i_/cm^−1^**
tsAMT(1-2)	224.8	208.6		1965i
tsAMT(1-5)	379.3	360.3		1509i
tsAMT(2-2′)	77.9	72.1		850i
tsAMT(2-4)	363.0	343.6		1429i
tsAMT(2′-4′)	405.8	387.1		805i
tsAMT(3′-3)	87.8	86.0		1008i
tsAMT(3-4′)	342.0	324.8		2032i
tsAMT(4-3′)	285.0	266.9		2046i
tsAMT(4-4′)	88.0	80.8		951i
tsAMT(5-4)	211.0	195.4		1969i

**Table 2:** Observed vibrational wavenumbers in an argon matrix (cm^−1^) compared with the calculated wavenumbers of the AMT1 tautomer of 2-amino-4-methylthiazole ^1^.

**B3LYP**			**MP2**			**AMT/Ar**		**Approximate Description ^4^**
**Harmonic Calc**	**Scaled ^2^ Calc**	**Int**	**Harmonic Calc**	**Scaled Calc**	**Int**	**Exper**	**Int**	
3672	3540	34 ^3^	3681	3508	38	3525.5 3515.0	w	ν_as_NH_2_
3564	3436	42	3563	3396	39	3419.5 3413.5	m	ν_s_NH_2_
3257	3140	1	3278	3124	1	n.o.	n.o.	νCH
3116	3004	15	3171	3022	9	3009.0	vw	ν_as_CH_3_
3081	2970	12	3151	3003	8	2971.5	vw	ν_as_CH_3_
3031	2922	27	3071	2927	22	2938.5	vw	ν_s_CH_3_
1639	1603	156	1641	1597	117	1600.5	vs	δNH_2_
1581	1546	69	1567	1525	41	1547.5 1544.5	m	Ring strech
1557	1523	109	1559	1517	92	1525.0	vs	Ring stretch
1483	1450	14	1499	1459	12	1448.0	w	δ_as_CH_3_
1476	1443	6	1492	1452	7	1383.0 1379.5	w	δ_as_CH_3_
1417	1386	10	1417	1379	10	1358.0	w	δ_s_CH_3_
1355	1326	117	1368	1331	70	1329.5 1326.5	vs	Ring stretch, δCH
1301	1272	36	1316	1280	57	1286.0 1282.5	vw	Ring stretch, ρNH_2_
1164	1139	7	1163	1132	5	1142.5	vw	δCH
1116	1092	20	1121	1091	28	1100.5 1094.5	vw	ρNH_2_
1064	1040	1	1065	1036	1	1037.5	vw	ρCH_3_
1015	992	6	1011	984	6	995.0	vw	ρCH_3_
976	954	14	976	950	14	965.5 963.0	vw	Ring def
844	825	7	876	852	7	851.5	vw	Ring def, νS-C
705	705	23	711	711	17	701.5	vw	γCH
675	675	8	678	678	198	692.5 687.0	m	Ring def, νS-C
657	657	31	699		8	677.0	vw	Ring def
614	614	31	628		20	657.0	vw	Ring breath
592	592	37	597		27	629.0	vw	Ring def
550	550	5	551	551	3	607.0	vw	Ring def
509	509	198	539	539	58	444.0	w	ωNH_2_
402	402	1	398	398	2			Skeletal def
299	299	14	255	255	27			τNH_2_
277	277	10	285	285	3			Skeletal def
267	267	8	272	272	4			Skeletal def
205	205	13	205	205	11			Skeletal def
144	144	0	146	146	0			τCH_3_

^1^ B3LYP/6-311++G(3df,3pd) and MP2/aug-cc-pVTZ methods were used in calculations. ^2^ The following scaling factors were used: 1.0/1.0 (below 750 cm^−1^), 0.978/0.973 (in the range 750–2500 cm^−^^1^), 0.964/0.953 (above 2500 cm^−1^) for B3LYP/MP2 calculations, respectively. ^3^ The IR calculated intensities expressed in km mol^−^^1^. ^4^ Abbreviations: ν, bond stretching; δ, bending, deformation in plane; ω, wagging; ρ, rocking; γ, out of plane bending, torsion; τ, twisting.

**Table 3:** Experimental and B3LYP/6-311++G(3df,3pd) calculated wavenumbers (cm^−1^) and intensities (km mol^−1^) of the photoproduct molecules observed after photolysis of AMT/Ar matrix.

fp1							Assignmt ^1^
fp1a			fp1s				
ν experim	ν harmon	ν anharm	ν experim	ν harmon		ν anharm	
2134 br	2217 (1338)		2171	2142 br	2221 (1332)	2182	ν_as_NCN
887.5	922 (443)		868	885.0	912 (406)	864	δNH
644 br	649 (24)		641		631 (15)	621	δNCN
**fp2**							**Assignmt**
**fp2a**			**fp2s**				
**ν experim**	**ν harm**	**ν anharm**	**ν experim**	**ν harm**	**ν anharm**		
2130 br	2221 (1185)		2158	2137 br	2219 (949)	2177	ν_as_NCN
	1282 (31)		1257	1263.0	1314 (26)	1281	δCH
1080.5	1085 (58)		1071	1057.5	1077 (30)	1058	ρCH_3_
1000.0	1025 (25)		998	998.5	1012 (27)	991	νC-C(H_3_)
873.0	932 (312)		855	898.0	927 (313)	898	δNH
	595 (53)		565	644 br	655 (33)	638	δCCN
**fp3**							**Assignmt**
**ν experim**	**ν harm**		**ν anharm**				
3443.5	3586 (91)		3424				νNH
2160 br	2233 (1035)		2193				ν_as_NCN
1373.5 1375.5	1426 (48)		1390				δNH
865.0	913 (217)		856				δNCN
**fp15**							**Assignmt**
**ν experim**	**fp15syn/syn**		**fp15anti/syn**		**fp15syn/anti**		
	**ν harm**	**ν anharm**	**ν harm**	**ν anharm**	**ν harm**	**ν aharm**	
1618.0	1653 (175)	1612	1652 (173)	1602	1677 (226)	1636	νC=N
1250.0	1255 (304)	1210	1268 (266)	1220	1262 (166)	1223	δNH
1103.0	1130 (68)	1101	1120 (52)	1096	1092 (47)	1066	νCN

^1^ Abbreviations: ν, bond stretching; δ, bending, deformation in plane; ρ, rocking.

**Table 4:** Wavenumbers and wavenumbers shifts (Δν = (ν_comp_ − ν_mon_)) (in cm^−1^) calculated and observed for the H_2_N–C≡N∙∙∙CH_3_–CSC–H complex in solid argon ^1^.

H_2_N–C≡N∙∙∙CH_3_–CSC–H						Assignment
Experimental		Calculated Harmonic				
		fp11-12a		fp11-12b		
ν	Δν	ν	Δν	ν	Δν	
3165 br	−235	3279	−268	3294	−253	ν_s_NH_2_
2256.0 2252.0	−8 −12	2335	−16	2333	−18	νC≡N
665.0	+251	695	+206	672	+183	ωNH_2_

^1^ In all calculations the B3LYP/6-311++G(3df,3pd) method was used. The experimental wavenumbers for the H_2_N-C≡N monomer were taken from refs. [24,25].

**Table 5:** Wavenumbers and wavenumbers shifts (Δν = (ν_comp_ − ν_mon_)) (in cm^−1^) calculated and observed for the H_2_N–C≡N∙∙∙CH_3_C≡CSH complex in solid argon ^1^.

H_2_N–C≡N∙∙∙CH_3_C≡CSH						Assignment
Experimental		Calculated Harmonic				
		fp11-13a		fp11-13b		
ν	Δν	ν	Δν	ν	Δν	
3475.0	−11	3615	−22	3607	−30	ν_as_NH_2_
3365 sh	−35	3493	−54	3471	−76	ν_s_NH_2_
2260.0	−4	2343	−8	2341	−10	νC≡N

^1^ In all calculations the B3LYP/6-311++G(3df,3pd) method was used. The experimental wavenumbers for the H_2_N–C≡N monomer were taken from ref. [24,25].

**Table 6:** Wavenumbers and wavenumbers shifts (Δν = (ν_comp_ − ν_mon_)) (in cm^−1^) calculated and observed for the H_2_N–C≡N∙∙∙S=C=C(H)CH_3_ complex in solid argon ^1^.

H_2_N–C≡N∙∙∙S=C=C(H)CH_3_				Assignment
Experimental		Calculated Harmonic		
		fp11-14		
ν	Δν	ν	Δν	
3466.0	−20	3610	−27	ν_as_NH_2_
3314.0	−86	3453	−94	ν_s_NH_2_
2267.0	+3	2342	−9	νC≡N
1777.0	0	1837	0	ν_as_C=C=S
638.0	+25	656	+26	γCH

^1^ In all calculations the B3LYP/6-311++G(3df,3pd) method was used. The experimental wavenumbers for the monomers were taken from refs. [24,25,26].

**Table 7:** Wavenumbers and wavenumbers shifts (Δν = (ν_comp_ − ν_mon_)) (in cm^−1^) calculated and observed for the H_3_C–C≡CH∙∙∙HN=C=N–SH complex in solid argon ^1^.

H_3_C–C≡CH∙∙∙HN=C=N–SH						Assignment
Experimental		Calculated Harmonic				
		fp22-25a		fp22-25b		
ν	Δν	ν	Δν	ν	Δν	
3337.0	−40	3464	−93	3555	−2	νNH
2117.5	−44.5 ^2^	2211	−1	2208	−4	ν_as_NCN
898 tentatively	+28	976	+31	949	+4	δNH
635	+4	675 668	+15 +2	668 660	+2 −6	δCH γCH

^1^ In all calculations the B3LYP/6-311++G(3df,3pd) method was used. The experimental wavenumbers for the propyne monomer were taken from ref. [27,28]; for the HN=C=N–SH monomer the anharmonic wavenumbers were used for Δν_exp_ calculations (Table S3). ^2^ The magnitude of this shift is explained in the text.

## Data Availability

The data presented in this study are available in this article.

## Supplementary Materials

The following supporting information can be downloaded online. Table S1: Selected structural parameters calculated for 2-amino-4-methylthiazole (AMT) tautomers at the B3LYP/6-311++G(3df,3pd) level of theory; Table S2: Harmonic wavenumbers, scaled wavenumbers (cm^−1^) and intensities (km mol^−1^) of 2-amino-4-methylthiazole photoproducts calculated at the B3LYP/6-311++G(3df,3pd) level of theory; Table S3: Anharmonic and harmonic wavenumbers (in cm^−1^) calculated for the fp1, fp2, fp3, fp15, fp25, fp23 molecules at the B3LYP/6-311++G(3df,3pd) level of theory; Table S4: Binding energy and CP-corrected energy (in kJ mol^−1^) calculated at B3LYP-D3(BJ) for the structures of complexes obtained after optimization at B3LYP/6-311++G(3df,3pd); Figure S1: B3LYP/6-311++G(3df,3pd) optimized structures of the tautomers of 2-amino-4-methylthiazole; Figure S2: B3LYP/6-311++G(3df,3pd) optimized structures of the transition states; Figure S3: Comparison of the AMT/Ar matrix spectrum with the spectra calculated for all of the tautomers of AMT molecule at the B3LYP/6-311++G(3df,3pd) level of theory; Figure S4: The 2300–2000 cm^−1^ region in the AMT/Ar matrix spectra during irradiation at wavelengths between 295 and 265 nm; Figure S5: Photoreaction pathways of AMT. Part 1; Figure S6: Photoreaction pathways of AMT. Part 2; Figure S7: 3600–2900 cm^−1^ (**a**), 2300–1200 cm^−1^ (**b**), 1200–420 cm^−1^ (**c**) regions of the spectra of AMT/Ar matrix after deposition (bottom spectra) and after 300 min irradiation at 265 nm of the deposited matrix (upper spectra); Figure S8: The 2135 cm^−1^ band in the AMT/Ar matrix spectra during irradiation at 265 nm.

## Sample Availability

Samples of the compounds are not available from the authors.
