# Supplementary material for: Structure, Spectra and Photochemistry of 2-Amino-4-Methylthiazole: FTIR Matrix Isolation and Theoretical Studies
Source: Molecules. 2022 Jun 17;27(12):3897. doi: 10.3390/molecules27123897 (PMC9227644; doi:10.3390/molecules27123897)
Supplement: Supplementary file 1 [file molecules-27-03897-s001.zip › molecules-1771168-supplementary.pdf]

Supplementary Materials

Structure, Spectra and Photochemistry of 2-amino-4-methylthiazole: FTIR Matrix Isolation and Theoretical Studies

Magdalena Pagacz-Kostrzewa [1], Daria Bumażnik [1], Stéphane Coussan [2,]* and Magdalena Sałdyka [1,]*

[1]*Faculty of Chemistry, University of Wroclaw, F. Joliot-Curie 14, 50-383 Wroclaw, Poland* [2]*Aix-Marseille Université, CNRS, PIIM, Marseille, France*

Table S1. Selected structural parameters calculated for 2-amino-4-methylthiazole (AMT) tautomers at the B3LYP/6-311++G(3df,3pd) level of theory. The bond distances are given in Å, the angles in degrees. E.g. see Scheme 1 for labels

Property	AMT1	AMT2	AMT2'	AMT3	AMT3'	AMT4	AMT4'	AMT5
r(S_1-C_2)	1.751	1.792	1.781	1.794	1.816	1.788	1.778	1.784
r(C_2-N_3)	1.297	1.381	1.388	1.414	1.407	1.378	1.387	1.280
r(N_3-C_4)	1.381	1.387	1.392	1.274	1.272	1.385	1.389	1.390
r(C_4-C_5)	1.357	1.341	1.340	1.512	1.515	1.514	1.512	1.534
r(C_5-S_1)	1.737	1.758	1.756	1.810	1.807	1.831	1.830	1.825
r(C_2-N_6)	1.374	1.271	1.271	1.261	1.261	1.266	1.266	1.357
r(C_4-C_9)	1.494	1.491	1.491	1.493	1.493	1.333	1.333	1.334
r(C_5-H_{13})	1.076	1.075	1.075	1.091	1.091	1.089	1.089	1.087
r(C_5-H_{14})				1.091	1.091	1.086	1.086	1.088
r(N_6-H_7)	1.008	-	1.016	1.018	-	-	1.017	1.007
r(N_6-H_8)	1.006	1.012	-	-	1.016	1.013	-	1.004
r(N_3-H_{15})						1.008	1.007	-
r(C_9-H_{10})	1.091	1.092	1.093	1.092	1.092	-	-	-
r(C_9-H_{11})	1.091	1.092	1.093	1.086	1.086	1.081	1.081	1.079
r(C_9-H_{12})	1.088	1.088	1.087	1092	1.092	1.078	1.078	1.080
ϕ(S_1-C_2-N_3)	114.6	106.5	106.8	111.9	111.1	108.8	109.1	117.9
ϕ(C_2-N_3-C_4)	111.4	117.5	117.1	113.7	114.3	119.7	119.1	113.4
ϕ(N_3-C_4-C_5)	115.2	112.7	112.4	118.6	118.9	110.1	109.8	113.7
ϕ(C_4-C_5-S_1)	110.3	111.8	112.2	105.0	104.8	106.6	106.6	105.8
ϕ(C_5-S_1-C_2)	88.5	91.5	91.5	90.8	91.0	92.6	92.6	88.9
ϕ(N_3-C_4-C_9)	118.8	119.2	119.2	122.1	122.0	125.4	125.2	123.5
ϕ(N_3-C_2-N_6)	123.7	123.0	130.6	126.5	122.0	122.4	129.5	123.8
ϕ(S_1–C_2–N_6)	121.6	130.5	122.5	121.7	127.0	128.8	121.4	118.2
θ($S_1C_2N_6H_7$)	-166.8	-	-180.0	180.0	-	-	-178.6	-170.6
θ($S_1C_2N_6H_8$)	-30.2	0.0	-	-	0.0	-1.6	-	-21.2
θ($N_6C_2N_3C_4$)	176.7	180.0	180.0	-180.0	180.0	174.8	-174.1	176.8
θ($C_2N_3C_4C_9$)	-180.0	-180.0	-180.0	-180.0	-180.0	-168.0	166.0	-176.0
θ($N_3C_4C_5H_{13}$)	179.2	180.0	-180.0	-120.4	-120.2	-135.3	-101.2	-123.3
θ($N_3C_4C_5S_1$)	-0.6	0.0	0.0	0.0	0.0	-15.3	17.4	-6.2

θ($S_1C_2N_3C_4$)	0.0	0.0	0.0	0.0	0.0	-4.4	5.3	-0.5
θ($S_1C_5C_4C_9$)	179.8	180.0	180.0	180.0	180.0	166.2	-164.0	174.4
θ($S_1C_2N_3H_{15}$)	-	-	-	-	-	-173.2	170.7	-

Table S2. Harmonic wavenumbers, scaled wavenumbers[a] (cm^{-1}) and intensities ($km\ mol^{-1}$) of 2-amino-4-methylthiazole photoproducts calculated at the B3LYP/6-311++G(3df,3pd) level of theory.

AMTfp1a			AMTfp1s			AMTfp2a			AMTfp2s			AMTfp3		
$\tilde{\nu}_h$	$\tilde{\nu}_{scal.}$	I	$\tilde{\nu}_h$	$\tilde{\nu}_{scal.}$	I	$\tilde{\nu}_h$	$\tilde{\nu}_{scal.}$	I	$\tilde{\nu}_h$	$\tilde{\nu}_{scal.}$	I	$\tilde{\nu}_h$	$\tilde{\nu}_{scal.}$	I
3571	3443	69	3579	3450	79	3584	3455	89	3591	3461	82	3586	3457	91
3203	3087	3	3218	3102	5	3122	3010	13	3126	3014	10	3203	3087	1
3119	3007	8	3117	3005	14	3116	3003	13	3117	3005	18	3134	3021	11
3072	2961	11	3063	2952	14	3088	2977	13	3077	2967	22	3113	3001	12
3023	2914	16	3017	2909	29	3058	2948	5	3045	2935	15	3102	2990	10
2686	2589	0	2694	2597	1	3041	2931	13	2957	2850	15	3036	2927	19
2217	2168	1339	2221	2172	1332	2221	2172	1185	2219	2170	949	2233	2184	1035
1665	1629	18	1673	1636	30	1496	1463	4	1501	1468	5	1497	1464	2
1486	1453	8	1488	1455	8	1488	1456	7	1489	1456	8	1487	1454	5
1481	1448	3	1477	1445	8	1414	1383	21	1418	1387	13	1476	1443	7
1424	1392	14	1430	1399	10	1401	1370	14	1410	1379	18	1426	1395	48
1416	1385	3	1418	1387	1	1380	1350	23	1363	1333	32	1417	1386	2
1312	1283	5	1291	1263	24	1333	1303	26	1314	1285	26	1282	1253	16
1139	1114	43	1154	1128	19	1282	1253	31	1279	1251	2	1204	1177	46
1062	1039	1	1065	1042	1	1167	1142	6	1170	1144	14	1089	1065	26
1027	1004	18	1051	1028	21	1106	1082	22	1105	1080	30	1075	1051	12
966	945	11	970	949	14	1085	1061	58	1077	1054	30	990	969	2
922	902	444	912	892	406	1025	1002	25	1012	990	21	943	922	12
855	836	16	856	837	48	932	912	313	927	907	313	924	904	52
847	828	42	806	788	19	893	873	6	892	873	3	913	893	237
768	751	16	746	746	36	862	843	18	854	835	15	777	760	32
649	649	24	631	631	15	835	817	7	759	742	19	656	656	27
579	579	42	606	606	31	644	644	17	655	655	33	614	614	81
489	489	35	513	513	26	595	595	53	615	615	15	593	593	15
447	447	4	505	505	5	483	483	55	524	524	20	554	554	9
423	423	50	421	421	72	438	438	17	452	452	53	476	476	39
360	360	1	368	368	1	349	349	13	426	426	27	405	405	43
266	266	9	251	251	6	343	343	17	336	336	2	379	379	18
232	232	1	208	208	9	293	293	6	250	250	5	331	331	8
154	154	16	189	189	5	233	233	1	234	234	2	286	286	3
127	127	22	159	159	2	143	143	3	129	129	7	233	233	0
112	112	2	98	98	1	78	78	4	98	98	1	107	107	1
63	63	2	83	83	11	21	21	3	62	62	7	31	31	8

AMTfp4			AMTfp5			AMTfp6			AMTfp7			AMTfp8		
$\tilde{\nu}_h$	$\tilde{\nu}_{scal.}$	I	$\tilde{\nu}_h$	$\tilde{\nu}_{scal.}$	I	$\tilde{\nu}_h$	$\tilde{\nu}_{scal.}$	I	$\tilde{\nu}_h$	$\tilde{\nu}_{scal.}$	I	$\tilde{\nu}_h$	$\tilde{\nu}_{scal.}$	I
3604	3474	40	3707	3574	51	3704	3570	69	3701	3568	51	3691	3558	48
3198	3083	6	3582	3453	20	3581	3452	108	3584	3455	52	3575	3446	68
3117	3005	14	3132	3020	16	3129	3017	9	3105	2993	23	3247	3130	3
3116	3004	25	3119	3007	11	3075	2964	3	3102	2991	11	3157	3043	0
3077	2967	12	3080	2969	13	3027	2918	1	3040	2930	23	3094	2983	3
3027	2918	24	3025	2916	29	2533	2442	74	3033	2924	16	2663	2567	3
1658	1621	120	1787	1748	170	1712	1674	497	1685	1648	399	1764	1725	3
1626	1590	19	1700	1662	357	1597	1562	27	1604	1569	5	1657	1621	527
1483	1451	13	1627	1591	28	1581	1546	60	1493	1460	7	1603	1567	88
1469	1437	7	1485	1453	6	1462	1429	9	1489	1456	6	1443	1411	32

1413	1381	3	1473	1440	6	1454	1422	49	1443	1411	12	1410	1379	72
1407	1376	13	1428	1396	7	1396	1365	45	1408	1377	7	1230	1203	27
1371	1341	73	1401	1370	8	1317	1288	105	1349	1319	38	1188	1162	30
1297	1269	9	1256	1229	68	1202	1175	54	1292	1264	132	1158	1133	1
1165	1139	14	1180	1155	27	1152	1127	29	1229	1202	20	1105	1081	1
1092	1068	39	1148	1123	73	1037	1015	4	1137	1112	17	1023	1001	23
1066	1043	3	1072	1048	11	982	960	6	1103	1079	11	957	936	20
1040	1018	5	1044	1021	2	944	924	43	1067	1043	12	908	888	6
944	923	2	961	940	7	927	907	45	1056	1033	5	865	846	51
921	901	8	938	918	14	805	787	31	918	897	12	832	813	6
838	819	4	721	721	7	740	740	13	851	833	14	776	758	3
789	772	8	645	645	6	599	599	2	767	750	6	728	728	0
748	748	15	607	607	3	564	564	10	690	690	4	666	666	5
710	710	5	563	563	4	531	531	10	579	579	0	606	606	19
640	640	2	506	506	2	498	498	7	519	519	6	528	528	3
507	507	22	382	382	4	486	486	2	479	479	3	483	483	33
477	477	31	326	326	24	380	380	167	423	423	10	452	452	53
422	422	9	275	275	123	338	338	17	329	329	166	380	380	193
338	338	10	247	247	17	245	245	9	296	296	66	299	299	2
261	261	31	193	193	4	191	191	2	212	212	3	215	215	10
232	232	32	163	163	0	160	160	2	202	202	1	180	180	2
179	179	4	105	105	1	128	128	3	172	172	2	141	141	7
149	149	3	71	71	8	75	75	14	87	87	7	123	123	8
**AMTfp9a**			**AMTfp9s**			**AMTfp10a**			**AMTfp10s**			**AMTfp15**		
$\tilde{\nu}_h$	$\tilde{\nu}_{scal.}$	I	$\tilde{\nu}_h$	$\tilde{\nu}_{scal.}$	I	$\tilde{\nu}_h$	$\tilde{\nu}_{scal.}$	I	$\tilde{\nu}_h$	$\tilde{\nu}_{scal.}$	I	$\tilde{\nu}_h$	$\tilde{\nu}_{scal.}$	I
3717	3583	48	3696	3563	37	3716	3583	69	3672	3540	55	3518	3391	7
3584	3455	56	3582	3453	30	3580	3451	54	3544	3416	30	3324	3204	18
3255	3137	2	3252	3135	2	3239	3122	7	3239	3122	7	3121	3008	6
3160	3046	2	3158	3044	2	3166	3052	2	3151	3038	3	3093	2982	4
3096	2985	14	3130	3017	25	3148	3035	2	3145	3031	3	3030	2921	15
3093	2982	22	3092	2981	15	3129	3017	21	3131	3018	21	2675	2579	2
1727	1689	585	1739	1701	416	1646	1610	254	1657	1620	186	1920	1878	14
1635	1599	15	1643	1607	50	1626	1590	162	1634	1598	39	1653	1617	175
1616	1580	101	1617	1582	43	1580	1545	1	1584	1549	53	1483	1450	7
1456	1424	14	1463	1431	11	1448	1416	9	1448	1416	7	1463	1431	12
1397	1367	56	1424	1393	42	1392	1361	55	1384	1353	92	1403	1372	5
1333	1304	37	1332	1303	36	1363	1333	112	1360	1330	11	1255	1227	304
1320	1291	36	1310	1282	59	1324	1295	20	1331	1302	41	1130	1105	68
1296	1268	153	1263	1235	61	1238	1211	12	1210	1183	8	1059	1036	3
1137	1112	33	1109	1085	80	1099	1075	104	1100	1075	126	1042	1019	43
1101	1077	83	1091	1067	27	1074	1050	6	1079	1055	0	1022	999	32
1001	979	3	991	969	6	1010	988	47	1019	997	41	944	924	1
951	930	27	945	925	13	967	946	12	948	927	12	914	893	11
932	912	43	934	913	45	902	882	18	915	895	29	861	842	67
892	872	0	893	873	0	886	867	73	912	892	33	841	822	57
838	819	10	806	788	17	843	824	11	900	880	7	753	736	33
709	709	5	739	739	3	756	740	2	749	749	7	700	700	2
604	604	3	663	663	14	607	607	15	676	676	4	649	649	37
598	598	4	652	652	5	598	598	4	605	605	9	540	540	4
579	579	37	525	525	17	544	544	2	549	549	5	462	462	8
512	512	6	509	509	5	496	496	3	498	498	7	408	408	3
391	391	0	417	417	49	433	433	11	417	417	152	344	344	4
321	321	236	399	399	179	293	293	50	372	372	6	269	269	9
273	273	9	295	295	2	252	252	102	292	292	8	251	251	22
212	212	3	197	197	1	245	245	6	250	250	3	241	241	4
146	146	4	156	156	4	207	207	29	181	181	3	159	159	0
116	116	4	144	144	2	130	130	2	120	120	1	137	137	4
107	107	8	51	51	8	78	78	1	72	72	0	41	41	2
**AMTfp16**			**AMTfp17**			**AMTfp18**			**AMTfp19**			**AMTfp20**		

$\tilde{\nu}_h$	$\tilde{\nu}_{scal.}$	I	$\tilde{\nu}_h$	$\tilde{\nu}_{scal.}$	I	$\tilde{\nu}_h$	$\tilde{\nu}_{scal.}$	I	$\tilde{\nu}_h$	$\tilde{\nu}_{scal.}$	I	$\tilde{\nu}_h$	$\tilde{\nu}_{scal.}$	I
3528	3401	30	3712	3578	55	3706	3573	49	3670	3538	47	3726	3592	76
3252	3135	1	3569	3441	54	3573	3445	48	3542	3414	29	3619	3489	53
3161	3047	3	3342	3222	22	3258	3141	1	3281	3163	5	3591	3462	43
3129	3017	11	3113	3001	4	3179	3064	8	3063	2953	50	3292	3174	14
3079	2969	9	3079	2969	5	3165	3051	0	2998	2890	79	3091	2980	37
3029	2920	11	3026	2917	14	3089	2978	19	2675	2579	2	3027	2918	90
1822	1781	345	1945	1902	6	1842	1802	168	1842	1801	126	1843	1803	164
1679	1642	25	1613	1577	206	1625	1589	166	1659	1622	418	1636	1600	189
1492	1459	12	1478	1445	7	1494	1461	5	1607	1572	241	1532	1498	231
1476	1444	11	1469	1436	11	1450	1418	27	1511	1478	19	1515	1481	156
1444	1412	4	1410	1379	3	1375	1345	349	1311	1283	158	1417	1386	156
1412	1381	10	1388	1357	233	1348	1319	380	1179	1153	9	1307	1278	226
1278	1250	66	1272	1244	110	1218	1191	39	1118	1093	1	1193	1167	51
1166	1140	170	1084	1060	66	1148	1122	2	1091	1067	2	1109	1085	1
1093	1069	79	1058	1034	0	1106	1082	4	1059	1036	51	1097	1073	35
1074	1050	4	1042	1019	19	1075	1051	5	1031	1008	6	1082	1058	47
1019	997	1	1007	985	49	1024	1002	1	999	977	2	1018	996	1
942	921	2	905	885	55	971	950	34	962	941	19	1010	988	4
925	905	42	896	876	16	918	898	57	947	926	17	1000	978	114
846	827	21	744	744	3	879	860	49	793	776	37	861	842	6
767	750	45	703	703	3	809	792	3	725	725	49	782	765	6
738	738	9	653	653	22	735	735	2	701	701	1	650	650	122
679	679	14	600	600	1	674	674	4	659	659	3	647	647	0
623	623	36	576	576	7	637	637	3	536	536	145	624	624	7
549	549	4	484	484	5	531	531	16	491	491	6	532	532	0
503	503	4	413	413	10	502	502	1	432	432	89	480	480	8
425	425	8	388	388	60	414	414	3	411	411	9	388	388	2
408	408	4	363	363	105	365	365	5	394	394	0	370	370	14
322	322	11	309	309	7	343	343	5	311	311	4	335	335	3
199	199	5	230	230	12	327	327	178	217	217	19	201	201	111
180	180	1	171	171	1	190	190	8	163	163	1	164	164	8
156	156	1	127	127	3	124	124	5	133	133	5	100	100	3
71	71	1	37	37	5	74	74	3	64	64	2	61	61	18

AMTfp12			AMTfp13			AMTfp14			AMTfp22			AMTfp26		
$\tilde{\nu}_h$	$\tilde{\nu}_{scal.}$	I	$\tilde{\nu}_h$	$\tilde{\nu}_{scal.}$	I	$\tilde{\nu}_h$	$\tilde{\nu}_{scal.}$	I	$\tilde{\nu}_h$	$\tilde{\nu}_{scal.}$	I	$\tilde{\nu}_h$	$\tilde{\nu}_{scal.}$	I
3306	3187	17	3082	2971	6	3141	3028	5	3473	3348	64	3198	3083	1
3102	2990	6	3077	2966	8	3117	3004	12	3087	2976	6	3198	3083	1
3081	2970	5	3023	2914	31	3071	2961	15	3087	2976	6	3127	3014	0
3024	2915	17	2637	2542	3	3024	2915	40	3028	2919	17	3123	3010	2
1890	1848	11	2289	2238	0	1836	1796	333	2222	2173	10	2041	1996	92
1477	1444	10	1482	1449	9	1494	1461	5	1479	1446	9	1486	1453	0
1464	1432	12	1474	1442	8	1484	1451	6	1479	1446	9	1429	1398	4
1407	1376	1	1417	1385	3	1409	1378	20	1417	1386	0	1110	1086	0
1058	1035	16	1081	1057	1	1281	1252	28	1058	1035	0	1021	998	1
1053	1030	1	1056	1033	0	1082	1058	0	1058	1035	0	1021	998	1
959	938	13	1051	1028	2	1049	1026	1	941	920	0	884	864	0
897	877	14	975	953	14	1011	989	25	666	666	48	876	857	55
668	668	30	570	570	0	781	764	1	666	666	48	876	857	55
645	645	9	397	397	0	630	630	28	346	346	10	373	373	8
436	436	3	396	396	0	556	556	8	346	346	10	373	373	8
297	297	9	183	183	11	393	393	9						
260	260	1	173	173	7	177	177	0						
188	188	0	27	27	10	142	142	0						

AMTfp11			AMTfp23			AMTfp24			AMTfp25			AMTfp27		
$\tilde{\nu}_h$	$\tilde{\nu}_{scal.}$	I	$\tilde{\nu}_h$	$\tilde{\nu}_{scal.}$	I	$\tilde{\nu}_h$	$\tilde{\nu}_{scal.}$	I	$\tilde{\nu}_h$	$\tilde{\nu}_{scal.}$	I	$\tilde{\nu}_h$	$\tilde{\nu}_{scal.}$	I
3637	3506	66	3706	3573	68	3528	3401	30	3557	3429	66	3739	3605	69
3547	3419	40	3578	3449	58	3443	3319	29	2673	2576	0	3733	3599	333

2351	2299	117	1831	1791	224	1839	1798	332	2212	2164	900	2236	2186	883
1630	1594	40	1620	1585	47	1169	1143	259	1356	1326	0	1318	1289	0
1192	1166	0	1245	1217	86	1117	1093	20	1028	1005	16	861	842	62
1095	1071	10	1090	1066	5	1050	1027	37	945	924	269	695	695	0
563	563	142	682	682	14	917	897	76	678	678	20	614	614	5
489	489	82	561	561	6	801	783	69	603	603	59	553	553	15
412	412	0	513	513	11	623	623	66	589	589	23	527	527	595
			393	393	8	600	600	4	465	465	44			
			327	327	5	471	471	6	196	196	18			
			243	243	211	393	393	14	176	176	5			

AMTfp21			AMTfp28			AMTfp29			AMTfp30			AMTfp31		
$\tilde{\nu}_h$	$\tilde{\nu}_{scal.}$	I	$\tilde{\nu}_h$	$\tilde{\nu}_{scal.}$	I	$\tilde{\nu}_h$	$\tilde{\nu}_{scal.}$	I	$\tilde{\nu}_h$	$\tilde{\nu}_{scal.}$	I	$\tilde{\nu}_h$	$\tilde{\nu}_{scal.}$	I
3623	3493	23	3477	3352	8	3475	3350	2	3124	3011	8	3223	3107	4
3529	3402	25	3450	3326	2	3467	3342	4	3095	2983	4	3104	2993	11
3291	3173	6	3234	3118	3	3141	3028	7	3071	2961	12	3076	2965	3
3149	3035	5	3105	2993	11	3129	3016	5	3058	2948	7	3073	2963	17
3095	2983	36	3059	2949	11	3086	2975	8	3048	2938	22	3069	2959	9
3029	2920	77	3013	2905	28	3022	2913	27	3021	2912	28	3020	2912	23
1818	1778	65	1676	1639	2	1830	1790	385	1827	1787	433	1684	1647	0
1643	1607	74	1488	1455	16	1497	1464	9	1676	1639	30	1669	1633	34
1520	1486	15	1474	1442	9	1483	1450	11	1492	1459	4	1487	1454	14
1451	1419	80	1423	1392	18	1443	1411	28	1485	1452	13	1478	1445	71
1277	1249	104	1414	1383	1	1402	1371	6	1470	1438	67	1473	1440	9
1262	1234	16	1334	1305	8	1379	1349	5	1456	1424	40	1417	1386	1
1212	1185	3	1278	1250	35	1279	1250	38	1411	1380	13	1285	1256	8
1111	1087	6	1219	1192	14	1254	1227	3	1302	1273	13	1256	1228	1
1095	1071	12	1142	1117	24	1204	1177	55	1267	1239	35	1153	1128	9
1071	1047	37	1137	1112	90	1171	1145	41	1173	1148	18	1108	1084	9
1041	1018	34	1121	1096	46	1135	1110	105	1161	1136	41	1065	1042	11
1009	987	4	1059	1036	2	1091	1067	12	1048	1025	2	1013	990	13
995	973	30	997	975	5	1073	1050	59	1020	998	15	1004	982	1
914	893	4	900	880	74	1042	1020	6	975	954	10	977	955	17
770	753	10	837	819	6	944	923	1	821	803	13	840	821	39
744	744	328	827	809	5	844	826	45	787	770	26	810	792	24
708	708	35	778	761	20	788	771	0	766	749	5	780	763	10
615	615	22	718	718	6	697	697	9	605	605	7	714	714	18
514	514	57	598	598	0	576	576	8	566	566	2	561	561	5
476	476	5	567	567	4	548	548	3	485	485	5	540	540	10
446	446	3	523	523	0	407	407	1	335	335	8	472	472	13
420	420	16	353	353	1	381	381	3	317	317	4	335	335	11
340	340	43	320	320	3	249	249	3	185	185	9	266	266	1
264	264	32	228	228	1	210	210	3	158	158	3	235	235	7
164	164	6	211	211	0	128	128	0	149	149	1	186	186	3
125	125	4	186	186	1	124	124	1	130	130	3	144	144	1
94	94	0	138	138	0	62	62	1	53	53	2	110	110	2

AMTfp32			AMTfp33			AMTfp34			AMTfp35			AMTfp36		
$\tilde{\nu}_h$	$\tilde{\nu}_{scal.}$	I	$\tilde{\nu}_h$	$\tilde{\nu}_{scal.}$	I	$\tilde{\nu}_h$	$\tilde{\nu}_{scal.}$	I	$\tilde{\nu}_h$	$\tilde{\nu}_{scal.}$	I	$\tilde{\nu}_h$	$\tilde{\nu}_{scal.}$	I
3289	3171	3	3378	3256	3	3670	3538	27	3672	3540	34	3705	3572	59
3245	3128	3	3177	3063	2	3562	3434	34	3561	3432	40	3580	3451	93
3156	3042	4	3157	3044	4	3487	3361	7	3517	3390	26	3171	3057	6
3120	3008	14	3101	2989	16	3180	3066	2	3084	2973	4	3118	3006	4
3098	2987	6	3065	2955	11	3147	3034	63	3082	2971	7	3056	2946	19
3043	2934	9	3020	2911	25	3110	2998	8	3024	2915	25	3016	2907	44
1657	1620	14	1765	1726	99	2037	1992	20	2278	2228	2	1751	1713	2
1649	1613	3	1677	1640	1	1685	1648	268	1719	1681	208	1652	1616	461
1494	1461	28	1487	1455	13	1639	1603	105	1624	1588	71	1593	1558	104
1471	1439	9	1477	1445	7	1473	1441	3	1480	1447	9	1487	1454	6
1463	1431	80	1416	1384	0	1336	1307	182	1473	1440	9	1486	1453	6

1443	1412	17	1351	1322	10	1263	1235	41	1416	1385	2	1412	1381	0
1416	1385	10	1345	1316	2	1114	1089	9	1313	1284	290	1354	1324	204
1326	1297	31	1292	1263	18	1097	1073	15	1137	1112	72	1332	1303	24
1202	1176	149	1122	1097	41	1060	1037	122	1090	1066	42	1129	1105	3
1089	1065	25	1106	1082	26	1025	1002	1	1080	1056	7	1099	1075	5
1073	1050	1	1068	1045	1	899	879	46	1056	1032	1	1059	1036	1
1010	988	13	1021	999	33	894	875	17	1048	1025	2	1013	990	48
975	954	29	975	953	37	780	763	30	803	785	37	972	951	36
955	934	6	966	945	16	771	754	69	639	639	52	814	796	19
900	880	5	770	753	3	662	662	87	624	624	30	724	724	4
786	769	5	770	753	9	598	598	4	597	597	19	668	668	9
653	653	6	667	667	14	552	552	3	498	498	146	648	648	2
620	620	3	552	552	6	520	520	7	492	492	61	588	588	1
464	464	0	452	452	1	479	479	10	413	413	5	549	549	7
442	442	4	418	418	11	431	431	229	398	398	6	394	394	70
406	406	5	341	341	2	370	370	11	356	356	5	387	387	17
353	353	4	267	267	5	329	329	13	346	346	25	351	351	21
254	254	4	250	250	2	304	304	8	257	257	5	309	309	183
235	235	2	194	194	1	248	248	5	187	187	7	237	237	1
174	174	0	118	118	3	116	116	5	80	80	3	169	169	2
85	85	0	96	96	4	73	73	5	60	60	3	165	165	2
40	40	0	72	72	1	35	35	5	20	20	0	129	129	10
**AMTfp37**			**AMTfp38**			**AMTfp39**			**AMTfp40**			**AMTfp41**		
$\tilde{\nu}_h$	$\tilde{\nu}_{scal.}$	I	$\tilde{\nu}_h$	$\tilde{\nu}_{scal.}$	I	$\tilde{\nu}_h$	$\tilde{\nu}_{scal.}$	I	$\tilde{\nu}_h$	$\tilde{\nu}_{scal.}$	I	$\tilde{\nu}_h$	$\tilde{\nu}_{scal.}$	I
3719	3585	62	3688	3556	46	3524	3398	18	3664	3533	29	3706	3573	33
3579	3450	73	3569	3441	54	3464	3340	27	3552	3424	28	3594	3464	30
3155	3042	4	3222	3106	9	3252	3135	1	3200	3085	6	3155	3042	10
3114	3002	13	3161	3047	2	3192	3077	2	3164	3050	5	3122	3009	30
3076	2965	13	3135	3023	8	3154	3041	7	3079	2968	30	2977	2870	28
3027	2918	39	3089	2977	12	3091	2980	4	2956	2850	46	2947	2841	31
2114	2068	615	1693	1656	55	1673	1636	334	1717	1679	262	1835	1795	356
1621	1586	230	1673	1636	307	1646	1610	23	1658	1622	3	1720	1682	511
1509	1476	9	1614	1579	20	1605	1570	46	1618	1583	56	1641	1605	38
1484	1451	6	1459	1427	17	1440	1409	66	1488	1455	3	1472	1440	6
1437	1405	113	1344	1315	138	1378	1348	13	1377	1347	13	1423	1392	6
1416	1385	15	1323	1294	33	1326	1297	1	1346	1316	5	1377	1347	61
1382	1352	259	1296	1267	37	1244	1217	32	1276	1248	109	1280	1252	81
1284	1256	185	1179	1154	2	1220	1193	1	1252	1224	39	1244	1216	98
1190	1163	1	1142	1117	18	1184	1158	195	1207	1180	2	1203	1177	4
1088	1064	4	1098	1073	4	1144	1119	43	1171	1145	4	1097	1073	10
1058	1035	1	1033	1010	51	1037	1014	11	1023	1000	12	1062	1038	9
964	943	46	1025	1003	5	1023	1000	36	995	973	39	1000	978	5
887	868	44	964	943	39	1006	983	33	957	936	6	966	945	25
741	741	6	930	910	19	947	926	0	906	886	23	943	923	13
719	719	15	769	753	28	890	871	132	818	800	21	848	830	35
665	665	15	713	713	7	783	766	23	720	720	11	670	670	9
596	596	7	681	681	10	759	742	4	665	665	34	649	649	16
587	587	1	627	627	17	599	599	71	644	644	85	569	569	11
531	531	7	477	477	47	556	556	5	607	607	23	537	537	10
446	446	2	455	455	95	514	514	1	547	547	111	397	397	12
394	394	3	427	427	84	492	492	5	506	506	32	274	274	33
326	326	169	363	363	8	411	411	8	438	438	10	330	330	70
229	229	2	354	354	23	274	274	1	410	410	18	324	324	138
180	180	1	321	321	34	193	193	1	390	390	19	227	227	3
134	134	0	261	261	8	169	169	0	354	354	9	133	133	1
102	102	3	116	116	5	160	160	0	235	235	12	57	57	5
33	33	5	104	104	1	130	130	0	95	95	1	42	42	2

Compfp11-12a			Compfp11-12b			Compfp11-13a			Compfp11-13b			Compfp11-14		
$\tilde{\nu}_h$	$\tilde{\nu}_{scal.}$	I	$\tilde{\nu}_h$	$\tilde{\nu}_{scal.}$	I	$\tilde{\nu}_h$	$\tilde{\nu}_{scal.}$	I	$\tilde{\nu}_h$	$\tilde{\nu}_{scal.}$	I	$\tilde{\nu}_h$	$\tilde{\nu}_{scal.}$	I
3598	3469	89	3597	3467	90	3615	3485	118	3607	3477	109	3610	3480	124
3308	3189	21	3310	3191	42	3493	3367	205	3471	3346	170	3453	3329	257
3279	3148	735	3294	3175	694	3094	2983	0	3083	2972	6	3139	3026	5
3104	2993	3	3107	2995	3	3087	2976	4	3080	2969	6	3122	3010	12
3088	2977	2	3087	2976	3	3029	2920	13	3024	2915	24	3090	2979	8
3024	2915	11	3028	2919	11	2639	2544	1	2616	2521	25	3032	2923	20
2335	2281	142	2333	2284	140	2343	2292	122	2341	2290	108	2342	2290	112
1891	1849	9	1895	1854	6	2279	2229	2	2276	2226	2	1837	1796	247
1632	1596	24	1637	1601	21	1621	1585	17	1622	1586	26	1634	1598	22
1477	1445	9	1476	1443	10	1481	1448	8	1482	1449	9	1489	1456	7
1464	1432	9	1463	1431	12	1474	1441	8	1475	1442	8	1485	1452	8
1413	1382	2	1408	1377	2	1420	1389	1	1418	1387	2	1409	1378	13
1227	1200	5	1225	1198	3	1204	1177	2	1216	1189	4	1273	1245	19
1106	1082	9	1103	1078	10	1100	1076	10	1100	1076	9	1205	1178	2
1060	1037	3	1056	1032	8	1075	1051	2	1076	1052	2	1103	1078	8
1055	1032	14	1054	1031	9	1059	1036	1	1056	1033	1	1080	1056	0
963	942	12	965	944	9	1053	1029	1	1052	1029	2	1052	1029	0
898	878	16	901	881	15	973	952	14	990	968	8	1005	983	24
695	695	124	686	686	39	608	608	139	635	635	133	776	759	2
676	676	28	672	682	117	569	569	0	569	569	0	656	656	32
642	642	9	636	636	10	515	515	16	517	517	14	594	594	138
532	532	3	531	531	3	420	420	1	422	422	0	555	555	8
440	440	3	444	444	5	401	401	0	405	405	1	521	521	14
436	436	6	438	438	2	399	399	0	397	397	1	429	429	1
320	320	5	315	315	36	219	219	11	244	244	37	391	391	7
298	298	63	294	294	31	202	202	36	223	223	30	226	226	40
265	265	1	261	261	3	170	170	9	191	191	14	175	175	0
198	198	4	181	181	5	140	140	13	181	181	4	148	148	0
153	153	33	148	148	28	91	91	7	96	96	11	111	111	23
87	87	2	86	86	0	52	52	2	55	55	9	75	75	0
71	71	7	40	40	11	43	43	7	47	47	11	61	61	8
53	53	6	38	38	11	21	21	11	40	40	7	50	50	1
39	39	12	22	22	4	16	16	10	33	33	1	31	31	13

Compfp22-23a			Compfp22-23b			Compfp22-24			Compfp22-25a			Compfp22-25b		
$\tilde{\nu}_h$	$\tilde{\nu}_{scal.}$	I	$\tilde{\nu}_h$	$\tilde{\nu}_{scal.}$	I	$\tilde{\nu}_h$	$\tilde{\nu}_{scal.}$	I	$\tilde{\nu}_h$	$\tilde{\nu}_{scal.}$	I	$\tilde{\nu}_h$	$\tilde{\nu}_{scal.}$	I
3647	3516	141	3695	3562	122	3529	3402	31	3466	3342	86	3555	3427	66
3506	3380	296	3482	3356	337	3450	3326	130	3464	3340	512	3468	3343	67
3463	3338	71	3464	3339	67	3444	3320	45	3095	2984	3	3095	2983	1
3095	2984	3	3095	2983	3	3087	2975	6	3094	2983	4	3091	2980	4
3089	2977	1	3088	2977	3	3086	2975	6	3033	2923	11	3031	2922	11
3028	2919	10	3027	2918	13	3028	2919	20	2678	2582	0	2641	2546	82
2211	2162	12	2208	2159	17	2219	2170	5	2215	2167	140	2216	2167	20
1831	1791	234	1827	1786	215	1842	1802	316	2211	2163	917	2208	2160	964
1630	1595	25	1626	1590	19	1479	1447	9	1479	1446	8	1481	1448	7
1480	1447	6	1478	1446	5	1479	1446	9	1478	1445	7	1478	1446	5
1477	1444	7	1478	1445	7	1417	1386	0	1419	1387	1	1419	1388	1
1420	1389	1	1424	1393	2	1165	1140	258	1357	1327	2	1354	1324	0
1246	1219	61	1260	1232	111	1117	1092	26	1062	1039	1	1063	1039	2
1106	1082	10	1099	1075	4	1058	1035	0	1059	1035	0	1059	1036	0
1063	1039	1	1064	1041	1	1057	1034	0	1027	1004	22	1054	1031	8
1058	1035	0	1059	1036	0	1051	1027	39	976	954	358	949	929	278
936	915	0	935	915	0	941	920	0	938	917	8	938	917	1
681	681	13	689	689	5	917	897	76	688	688	21	680	680	31
676	676	66	668	668	76	802	785	71	675	675	69	668	668	59

659	659	45	653	653	59	695	695	34	668	668	99	660	660	47
593	593	4	597	597	4	694	694	52	654	654	3	602	602	61
517	517	11	523	523	11	619	619	66	594	594	6	588	588	18
469	469	37	490	490	8	600	600	6	508	508	23	465	465	43
377	377	120	368	368	16	472	472	7	360	360	15	354	354	15
363	363	15	353	353	11	392	392	15	349	349	8	349	349	10
352	352	8	338	338	5	348	348	9	195	195	16	288	288	8
335	335	11	230	230	132	348	348	7	184	184	5	182	182	11
106	106	8	101	101	9	52	52	1	105	105	4	66	66	4
77	77	0	88	88	0	39	39	0	74	74	2	63	63	2
76	76	0	77	77	0	35	35	0	58	58	0	60	60	1
47	47	2	59	59	6	23	23	0	53	53	1	45	45	5
41	41	3	35	35	4	18	18	3	26	26	2	31	31	0
23	23	1	17	17	9	10	10	1	12	12	1	14	14	1

[a] The following scaling factors were used: 1.0 (below 750 cm^{-1}), 0.978 (in the range 750 - 2500 cm^{-1}), 0.964 (above 2500 cm^{-1})

Table S3. Anharmonic and harmonic wavenumbers (in cm^{-1}) calculated for the fp1, fp2, fp3, fp15, fp25, fp23 molecules at the B3LYP/6-311++G(3df,3pd) level of theory. The IR calculated intensities expressed in km mol^{-1}.

fp1s

Mode(Quanta)	E(harm)	E(anharm)	I(harm)	I(anharm)
1(1)	3579.037	3392.804	78.87965004	60.89664633
2(1)	3217.960	3089.359	4.57881029	4.32176559
3(1)	3117.260	2978.750	13.74120208	12.65420903
4(1)	3062.612	2918.393	13.85564414	15.90955940
5(1)	3017.155	2931.480	29.45786749	32.35640097
6(1)	2693.651	2457.346	0.76080442	1.27506352
7(1)	2220.583	2182.067	1332.16112647	1179.75039275
8(1)	1672.529	1623.091	30.08139650	19.41823772
9(1)	1487.714	1451.866	7.93513538	6.58501319
10(1)	1477.131	1443.350	7.99217670	4.04266935
11(1)	1430.125	1403.450	9.77005549	9.19818070
12(1)	1417.917	1386.302	0.96432053	0.98974434
13(1)	1291.281	1270.711	24.43506090	19.17591972
14(1)	1153.525	1126.196	18.68860530	16.60794085
15(1)	1065.250	1043.510	0.95161737	1.19399670
16(1)	1050.755	1035.206	20.54866945	4.75382240
17(1)	969.860	942.979	14.17423717	13.75467410
18(1)	911.963	863.714	406.28646750	306.05944352
19(1)	855.881	840.250	47.92019736	64.75408297
20(1)	805.822	799.249	18.85861358	16.80008083
21(1)	746.219	732.737	35.78032951	27.21522965
22(1)	630.715	621.447	15.13232213	9.67126845
23(1)	606.227	594.787	31.36184063	18.95645036
24(1)	512.737	507.958	26.31354417	31.62609754
25(1)	505.268	503.449	4.63374673	2.14317342
26(1)	421.497	398.940	71.89799027	70.09548601
27(1)	368.274	367.535	1.11067806	0.53044877
28(1)	250.723	196.609	6.20785954	32.42692743
29(1)	207.834	27.344	8.99416334	2.75601133
30(1)	188.493	185.678	4.89010505	3.98733226
31(1)	158.839	15.762	1.95660053	18.87152060
32(1)	98.089	97.908	0.95417152	0.79447876
33(1)	82.640	88.098	10.74112011	12.49386459

fp1a

Mode(Quanta)	E(harm)	E(anharm)	I(harm)	I(anharm)
1(1)	3571.175	3409.900	68.91882044	55.53130826
2(1)	3202.701	3055.854	3.39888014	3.47034305
3(1)	3119.056	2987.162	7.81666071	6.11776138
4(1)	3071.863	2928.115	10.52305008	13.02554389
5(1)	3022.491	2927.888	15.51437393	18.62547306
6(1)	2685.679	2461.416	0.07168020	1.75894077
7(1)	2216.690	2171.390	1338.33162068	765.93869348
8(1)	1665.450	1617.228	18.50050867	21.31096734
9(1)	1486.110	1441.788	7.88978666	5.68464230
10(1)	1480.862	1437.902	2.81744542	3.49573430
11(1)	1423.529	1392.149	14.31575927	12.47440937
12(1)	1416.434	1386.359	2.66152837	85.42555816
13(1)	1312.259	1293.433	5.01457881	2.81188733
14(1)	1139.151	1114.747	42.82101323	23.12364169
15(1)	1062.105	1041.508	0.53638183	0.25094606
16(1)	1026.854	1008.942	17.73638306	7.20950022
17(1)	966.358	947.322	11.16516787	2.49928692
18(1)	922.103	868.490	443.49822167	346.43898915
19(1)	854.699	837.078	16.37561241	14.39907094
20(1)	846.977	825.064	41.74332414	36.44542576
21(1)	767.647	760.615	16.07906623	9.88254806
22(1)	649.137	640.843	23.76333207	18.30554449
23(1)	579.368	566.090	42.17498812	27.18958886
24(1)	489.023	488.953	34.53475529	33.79411489
25(1)	446.825	446.122	4.46131955	1.41176410
26(1)	423.138	413.704	49.90919040	57.81397598
27(1)	360.302	358.130	0.62578797	0.17871635
28(1)	265.855	248.392	9.43423989	9.98896651
29(1)	231.960	226.129	0.90686270	2.35992384
30(1)	154.044	94.553	16.42048729	192.52242781
31(1)	126.854	-29.542!	21.93289544	156.83659482
32(1)	111.728	112.136	2.12094702	95.62964612
33(1)	62.749	70.016	2.06589614	4.07193417

fp2s

Mode(Quanta)	E(harm)	E(anharm)	I(harm)	I(anharm)
1(1)	3590.521	3419.136	82.35195876	63.48529681
2(1)	3126.096	2977.955	10.29119343	12.45939141
3(1)	3117.269	2970.232	17.99768099	19.36834067
4(1)	3077.372	2926.979	22.22694845	32.98311203
5(1)	3044.499	2950.857	15.02425749	15.62876147
6(1)	2956.827	2801.725	14.72071221	18.35622606
7(1)	2218.893	2176.756	948.81236385	472.54628291
8(1)	1500.878	1459.162	4.58777723	2.95105664
9(1)	1488.524	1442.741	7.65666555	6.32154927
10(1)	1417.855	1394.035	12.74750399	41.92306027
11(1)	1409.953	1373.536	18.04787850	5.22442588
12(1)	1363.136	1328.286	32.39336807	15.33517305
13(1)	1314.116	1281.003	26.21590158	20.04658902
14(1)	1279.472	1246.575	1.77926262	1.53911624
15(1)	1170.134	1142.141	14.33292018	15.48022434
16(1)	1104.742	1080.240	30.35152750	22.87819004
17(1)	1077.264	1058.153	29.80642123	15.77095544
18(1)	1012.247	991.179	26.57848304	16.07452875
19(1)	927.286	898.397	313.11763014	216.79645660
20(1)	892.223	874.121	2.79078529	44.20758197
21(1)	853.749	846.573	15.44261126	20.03719668
22(1)	758.815	743.566	18.80927355	5.99829168
23(1)	654.507	638.439	33.06349360	21.37802944
24(1)	614.615	599.716	14.52607536	13.93580682
25(1)	523.976	515.228	19.99220371	22.00600867
26(1)	451.983	438.002	53.17163958	32.65679573
27(1)	426.271	413.619	26.57626073	57.91663210

28(1)	336.136	333.967	1.62707963	1.56861879
29(1)	250.123	236.725	4.56113589	7.13712376
30(1)	233.594	220.919	2.04623067	0.90060526
31(1)	128.512	125.250	6.65870366	6.10915931
32(1)	97.903	97.219	0.86373588	0.71731891
33(1)	62.456	57.913	6.72080629	6.77449826

fp2a

Mode(Quanta)	E(harm)	E(anharm)	I(harm)	I(anharm)
1(1)	3583.641	3409.742	88.63692916	52.41525687
2(1)	3122.084	2975.963	13.42069588	13.23245038
3(1)	3115.507	2970.545	12.69287710	20.12717061
4(1)	3087.769	2945.213	12.60592068	15.61472068
5(1)	3058.263	2910.661	4.57519496	5.09127978
6(1)	3040.829	2944.779	12.52661027	13.94686836
7(1)	2220.656	2158.380	1184.79255543	805.59322463
8(1)	1496.059	1456.247	3.51945191	1.98044953
9(1)	1488.278	1451.043	7.23698826	5.64133978
10(1)	1414.266	1380.604	20.50805815	14.87065015
11(1)	1400.667	1368.816	13.51466185	14.33033308
12(1)	1380.420	1357.769	23.12774867	4.19713481
13(1)	1332.541	1302.341	25.80824698	31.75356385
14(1)	1281.673	1256.753	31.04756064	19.95366226
15(1)	1167.437	1135.413	6.45330986	6.07310769
16(1)	1106.278	1080.416	22.32839105	25.42421471
17(1)	1085.120	1070.593	58.22526985	18.11359114
18(1)	1024.635	998.115	24.83132050	40.93440913
19(1)	932.168	855.334	312.61926954	204.76162946
20(1)	892.737	875.570	6.11573420	6.68315498
21(1)	861.696	852.155	18.49410870	16.55339045
22(1)	835.265	820.060	6.97799864	5.34799558
23(1)	644.244	634.759	17.06445734	18.12387869
24(1)	594.725	565.636	52.93291704	39.36631491
25(1)	483.084	459.255	55.37603093	44.93230658
26(1)	438.170	430.828	17.13492335	24.93502229
27(1)	349.326	349.650	13.43938876	1.51816069
28(1)	342.956	337.675	16.56576676	67.37432410
29(1)	292.667	291.536	6.19750106	5.03851633
30(1)	233.439	246.429	0.75014252	0.94823265
31(1)	143.491	140.246	2.92281473	3.19455623
32(1)	78.300	85.014	4.36114687	4.24238145
33(1)	21.239	14.622	2.57645555	3.16711322

fp3

Mode(Quanta)	E(harm)	E(anharm)	I(harm)	I(anharm)
1(1)	3586.411	3424.283	91.05821098	75.02715918
2(1)	3202.795	3051.307	1.24938673	3.25867462
3(1)	3134.210	2993.501	11.30807419	11.98776646
4(1)	3112.637	2989.965	11.98043818	15.00268718
5(1)	3102.072	2959.198	10.30438583	12.24172603
6(1)	3036.267	2938.206	19.24603174	21.41211523
7(1)	2233.284	2193.231	1035.24556025	878.17700968
8(1)	1497.027	1455.081	1.66431023	1.70713286
9(1)	1486.821	1448.698	5.16130830	4.11211158
10(1)	1475.588	1442.947	6.80809862	3.43104984
11(1)	1426.158	1390.174	47.89765264	34.75423613
12(1)	1416.691	1384.975	2.25813713	1.70406279
13(1)	1281.713	1253.875	16.27037631	10.70021636
14(1)	1203.627	1178.437	46.11435488	42.51479085
15(1)	1089.325	1067.537	25.96646479	18.32532389
16(1)	1075.021	1052.423	11.95049174	18.12093921
17(1)	990.354	971.929	2.22895760	2.88509299
18(1)	942.584	924.334	11.62133781	1.10368411
19(1)	924.503	902.129	51.78676321	24.50152174
20(1)	912.914	856.268	216.63441836	275.41154961
21(1)	776.733	758.334	31.76416883	21.00554929
22(1)	656.119	643.999	26.66957705	20.74323281

23(1)	614.493	600.363	80.71746364	28.23643049
24(1)	592.826	584.527	15.25594239	17.53523032
25(1)	554.067	545.826	9.01244729	16.73787795
26(1)	476.447	473.718	39.01145479	29.66709963
27(1)	404.748	403.905	42.79834158	40.94867885
28(1)	378.878	376.973	18.24109497	12.64289443
29(1)	331.277	332.945	7.61929691	10.86161721
30(1)	285.792	281.789	2.61315954	2.66948464
31(1)	233.340	235.042	0.14379310	0.12193763
32(1)	107.313	110.650	0.91660849	0.84469262
33(1)	30.605	83.837	7.96363468	0.39470294

fp15 syn/syn

Mode(Quanta)	E(harm)	E(anharm)	I(harm)	I(anharm)
1(1)	3517.919	3344.502	6.92131525	5.55894620
2(1)	3323.701	3182.641	17.76779502	10.39208355
3(1)	3120.606	2971.672	5.90153928	6.16384548
4(1)	3092.985	2947.312	4.08004477	6.33633944
5(1)	3030.177	2921.953	14.61738334	15.31156600
6(1)	2674.950	2677.458	1.54852413	2.25339274
7(1)	1920.295	1883.342	14.25270041	8.29597795
8(1)	1653.080	1611.659	175.10947089	92.46289622
9(1)	1482.674	1443.768	6.90864083	5.19913910
10(1)	1463.080	1415.057	12.36929973	10.43548304
11(1)	1403.126	1371.389	5.14159717	3.75494125
12(1)	1254.591	1209.565	303.85049520	192.15707373
13(1)	1129.866	1101.313	67.99661556	36.30147316
14(1)	1058.942	1031.085	3.12148380	0.70261764
15(1)	1042.335	1007.855	42.56209892	30.32371750
16(1)	1021.789	1000.227	32.47963517	35.69913539
17(1)	944.467	942.415	1.06782700	6.10939912
18(1)	913.546	905.929	11.24603797	3.73134216
19(1)	861.389	842.406	66.56858964	54.03164541
20(1)	840.541	820.918	57.09248518	50.72257135
21(1)	752.991	734.956	32.72940820	32.95334908
22(1)	699.597	684.461	2.21972356	2.09169322
23(1)	648.854	614.998	36.82731548	32.06643528
24(1)	539.981	517.412	3.61853539	3.21360546
25(1)	461.491	448.372	8.34281395	7.95305088
26(1)	408.371	404.975	2.52017543	1.99347825
27(1)	343.988	337.629	4.01261296	3.77262919
28(1)	269.500	332.706	8.55863733	9.37441858
29(1)	250.987	243.294	21.53645070	20.35073767
30(1)	241.384	240.583	3.62189169	3.09188458
31(1)	159.224	158.870	0.27777371	0.39442669
32(1)	136.537	140.412	4.44372174	4.41665632
33(1)	40.668	41.816	2.31017269	2.17909671

fp15 anti/syn

Mode(Quanta)	E(harm)	E(anharm)	I(harm)	I(anharm)
1(1)	3502.456	3335.923	7.22760934	5.40165302
2(1)	3321.282	3180.190	16.23077049	8.23792608
3(1)	3120.462	2973.533	5.52646810	5.67370990
4(1)	3093.446	2948.362	4.44792305	7.00604880
5(1)	3030.230	2921.024	15.18369620	15.64730400
6(1)	2684.213	2563.843	1.42903826	1.60998530
7(1)	1922.881	1887.289	14.58463265	5.45607222
8(1)	1651.704	1601.806	172.74132478	10.83346386
9(1)	1482.962	1442.348	7.12293457	2.26862182
10(1)	1463.379	1414.778	12.32178430	8.11779898
11(1)	1402.490	1371.539	5.02777113	4.32132462
12(1)	1267.681	1220.168	265.89102885	107.28341366
13(1)	1120.095	1096.462	51.77886020	29.84959449
14(1)	1057.704	1029.084	1.03675825	0.87408193
15(1)	1037.330	999.126	28.33806928	10.23399363
16(1)	1021.240	996.115	54.05345414	50.61281439
17(1)	929.315	901.434	7.32042131	6.39014418

18(1)	909.897	876.601	2.85847447	2.34749737
19(1)	859.829	837.930	37.30221365	34.66304881
20(1)	834.631	813.236	64.75487786	58.17726123
21(1)	763.733	745.049	30.23493746	28.45053413
22(1)	704.033	689.341	2.81831379	3.09760236
23(1)	648.914	623.800	37.63357673	31.89182468
24(1)	538.112	516.276	2.35963184	1.87954905
25(1)	457.707	446.146	9.64495531	9.93395609
26(1)	391.369	389.715	2.80594451	0.65969756
27(1)	344.967	339.380	6.48786424	6.35020488
28(1)	268.622	208.551	20.17123780	71.77434944
29(1)	249.527	227.539	13.81575919	8571.07308194
30(1)	246.460	213.807	6.22307913	19668.11335604
31(1)	157.716	154.311	0.30337803	0.95479937
32(1)	139.958	135.240	3.95747628	3.80577679
33(1)	43.481	41.044	2.19488678	2.26648024

fp15 syn/anti

Mode(Quanta)	E(harm)	E(anharm)	I(harm)	I(anharm)
1(1)	3453.415	3290.012	6.78479518	4.21935075
2(1)	3326.080	3186.870	20.44265269	15.77413742
3(1)	3117.367	2963.557	5.16527972	6.78939822
4(1)	3086.953	2937.471	2.85800925	3.31347712
5(1)	3029.617	2917.866	9.67044689	10.16011545
6(1)	2685.581	2674.461	1.31993165	1.10775521
7(1)	1932.464	1904.480	9.12885354	2.73622322
8(1)	1676.859	1636.061	226.21707617	119.69282341
9(1)	1477.720	1435.039	6.14940331	3.34699648
10(1)	1466.725	1424.962	10.01552701	5.27956560
11(1)	1408.346	1378.711	2.54401467	1.67604268
12(1)	1261.882	1222.690	166.35944150	63.96407348
13(1)	1091.820	1066.581	47.16831405	39.19881778
14(1)	1057.433	1034.275	5.03256371	3.21851436
15(1)	1027.269	998.133	22.35647881	16.13005766
16(1)	1015.238	982.460	26.81162947	8.14356328
17(1)	920.353	900.705	34.35376190	7.51036029
18(1)	901.087	885.592	23.59002938	28.84326822
19(1)	889.608	864.218	53.88052751	45.25764015
20(1)	874.287	858.827	61.22058502	79.73285359
21(1)	681.030	665.388	12.67388208	6.64655702
22(1)	669.443	652.691	57.50431010	53.66263210
23(1)	603.658	592.954	18.37496511	16.15401610
24(1)	520.381	513.131	17.14086653	13.38913913
25(1)	480.556	458.815	0.96509491	1.01426382
26(1)	426.347	422.663	4.12482964	1.80978360
27(1)	390.361	422.127	18.71586605	41.83826528
28(1)	359.785	384.401	10.02244450	9.71680901
29(1)	313.813	314.545	3.93768712	4.99622192
30(1)	218.341	215.924	1.18287583	0.95548861
31(1)	154.569	118.636	0.08415239	0.77011807
32(1)	127.748	129.864	1.79449674	1.58379244
33(1)	60.140	64.689	0.25009239	0.27846217

fp25

Mode(Quanta)	E(harm)	E(anharm)	I(harm)	I(anharm)
1(1)	3557.051	3376.794	66.14187454	18.93947281
2(1)	2672.662	2685.108	0.13780258	0.35438356
3(1)	2212.193	2162.602	900.27581087	788.47270947
4(1)	1356.253	1367.791	0.04381699	0.14773980
5(1)	1027.930	1028.936	15.91445753	12.53476176
6(1)	944.969	880.123	269.13221950	226.86249986
7(1)	677.933	667.467	19.52115846	13.95618915
8(1)	603.125	586.292	58.93044130	39.50241631
9(1)	589.450	579.274	22.75631703	43.68113758
10(1)	465.377	459.601	44.02735748	43.56862777
11(1)	195.915	313.024	17.85182495	13.57545402

```
  12(1)                 176.477    177.960       5.32650460       7.15282244
```

fp23

```
-----------------
  Mode(Quanta)          E(harm)    E(anharm)        I(harm)           I(anharm)
   1(1)                 3706.472   3575.446      68.07107671       67.43753187
   2(1)                 3578.221   3455.658      58.01284153       63.13329420
   3(1)                 1831.312   1800.362     224.13333213      214.13955244
   4(1)                 1620.427   1576.369      47.12081906       57.58070769
   5(1)                 1244.695   1200.474      85.50481242       81.04406848
   6(1)                 1089.723   1048.269       4.76949525        0.49575179
   7(1)                  681.890    666.999      13.83711720       14.51617098
   8(1)                  560.583    555.878       6.43145631        8.75743468
   9(1)                  512.809    502.094      10.78398790       14.18573782
  10(1)                  392.531    401.211       8.02284790      103.13211815
  11(1)                  326.806    306.196       5.34855324      562.02952901
  12(1)                  242.695   -642.867     211.39369430     3647.07214783
```

Table S4. Electronic energy and CP-corrected energy (in kJ mol^{-1}) calculated at B3LYP-D3(BJ) for the structures of complexes obtained after optimization at B3LYP/6-311++G(3df,3pd).

complex	ΔE	ΔE^{CP}
fp11-12a	-45.9	-44.4
fp11-12b	-39.8	-38.3
fp11-13a	-27.9	-26.9
fp11-13b	-30.9	-29.9
fp11-14	-29.8	-28.7
fp22-25a	-17.7	-17.0
fp22-25b	-16.2	-15.4

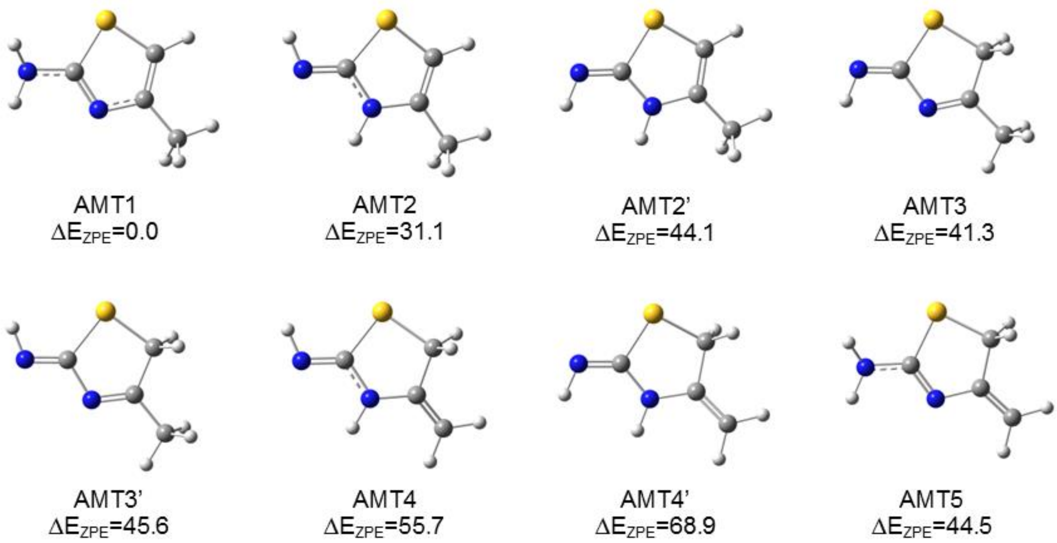

Figure S1. B3LYP/6-311++G(3df,3pd) optimized structures of the tautomers of 2-amino-4-methylthiazole.

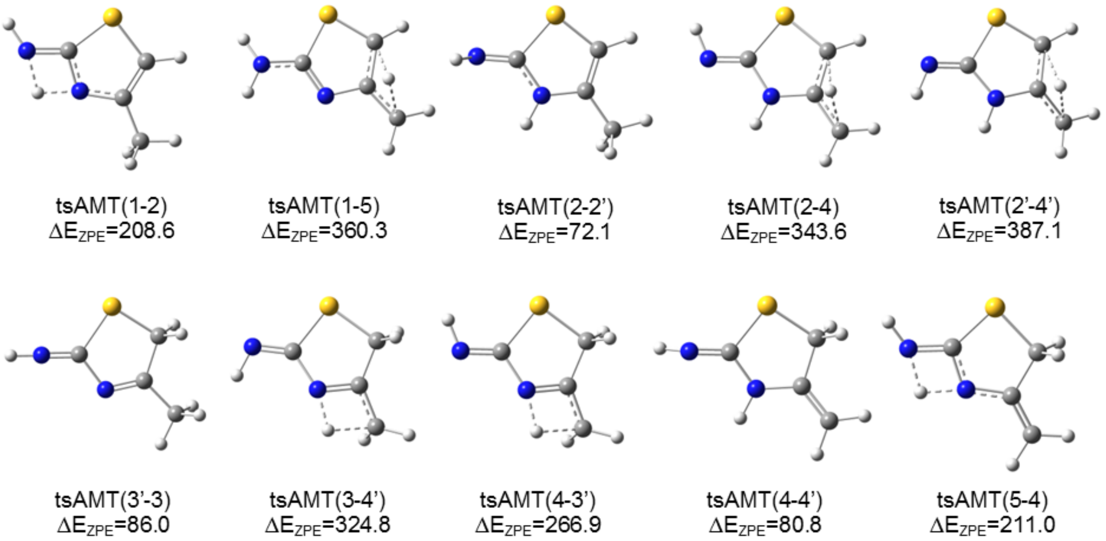

Figure S2. B3LYP/6-311++G(3df,3pd) optimized structures of the transition states.

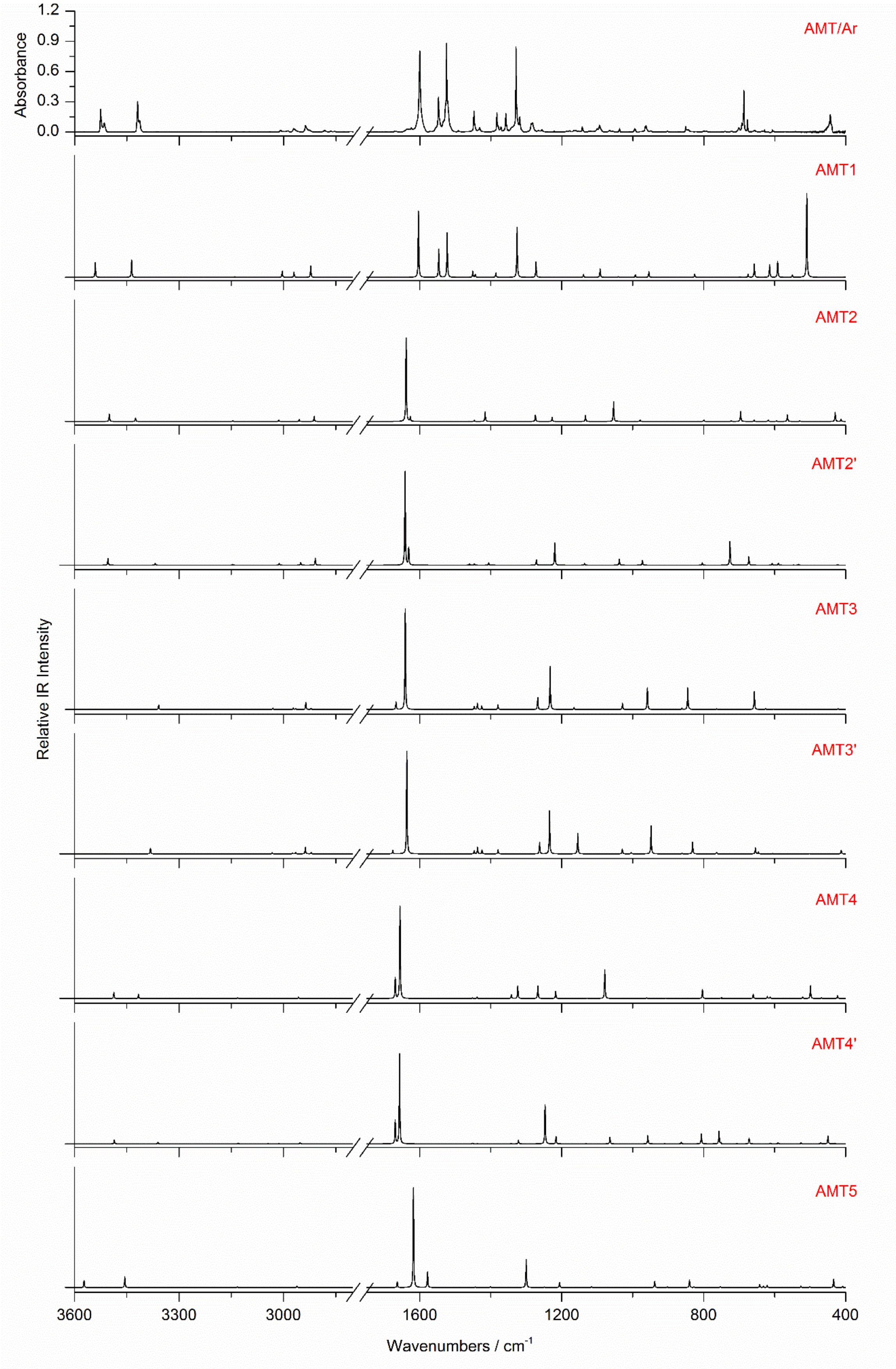

Figure S3. Comparison of the AMT/Ar matrix spectrum with the spectra calculated for all the tautomers of AMT molecule at the B3LYP/6-311++G(3df,3pd) level of theory.

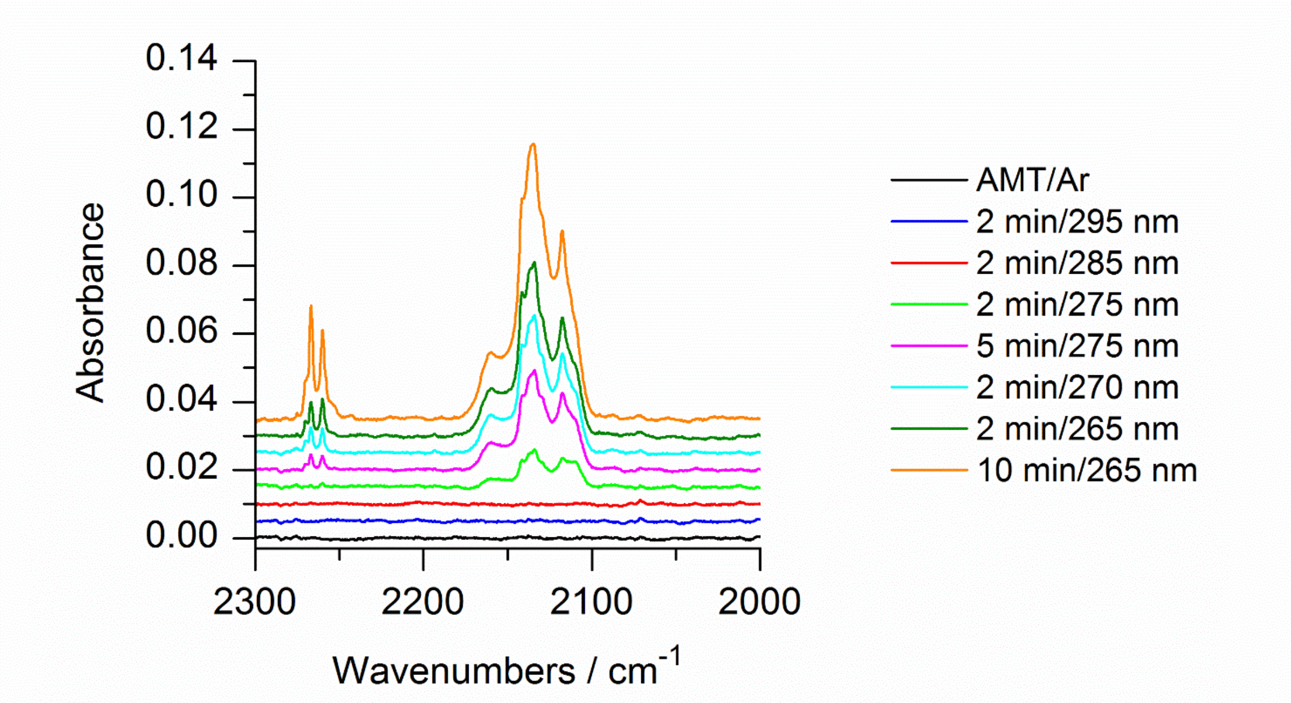

Figure S4. The 2300-2000 cm^{-1} region in the AMT/Ar matrix spectra during irradiation at wavelengths between 295 and 265 nm.

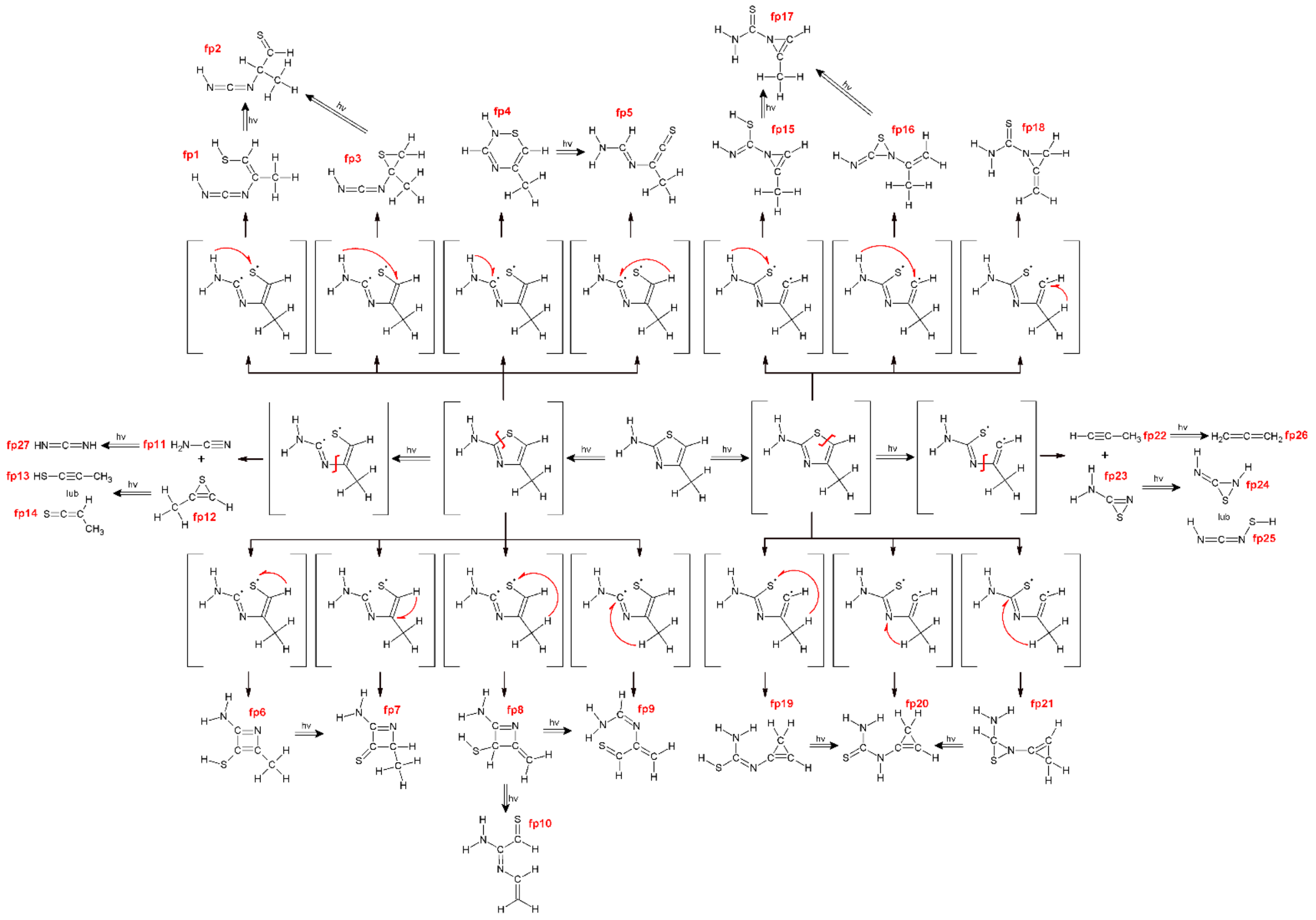

Figure S5. Photoreaction pathways of AMT. Part 1.

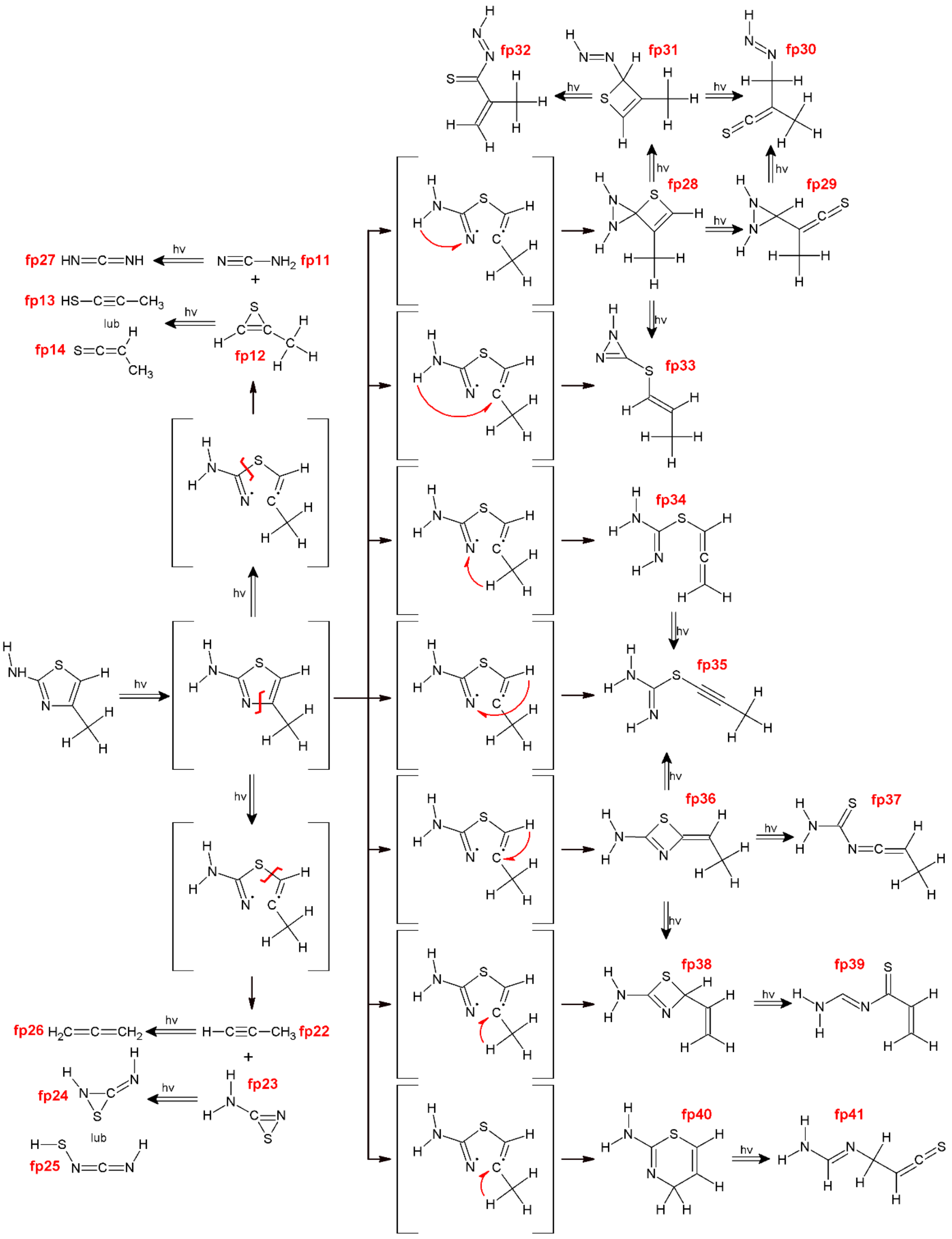

Figure S6. Photoreaction pathways of AMT. Part 2.

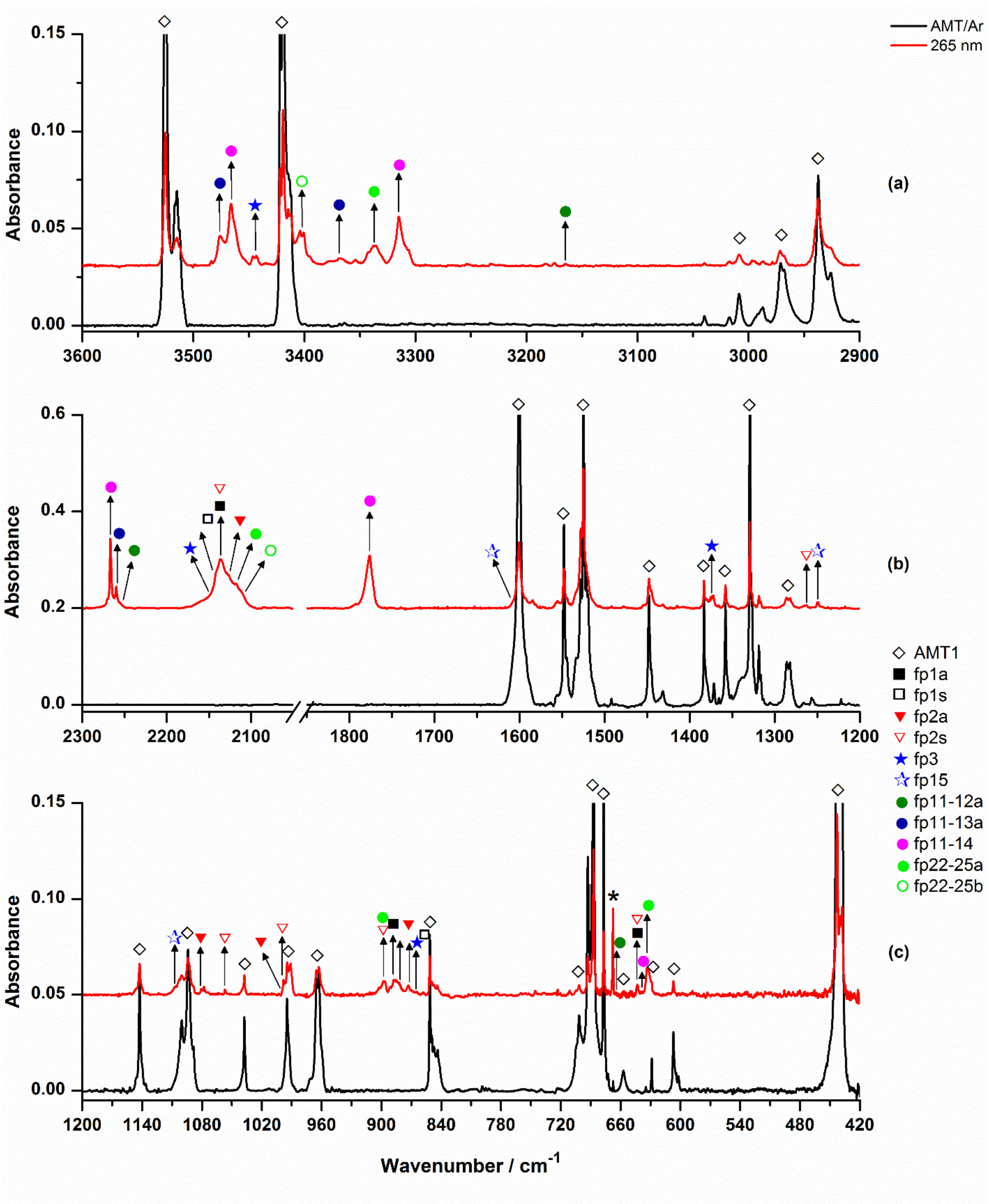

Figure S7. 3600-2900 cm^{-1} (A), 2300-1200 cm^{-1} (B), 1200-420 cm^{-1} (C) regions of the spectra of AMT/Ar matrix after deposition (bottom spectra) and after 300 min irradiation at 265 nm of the deposited matrix (upper spectra).

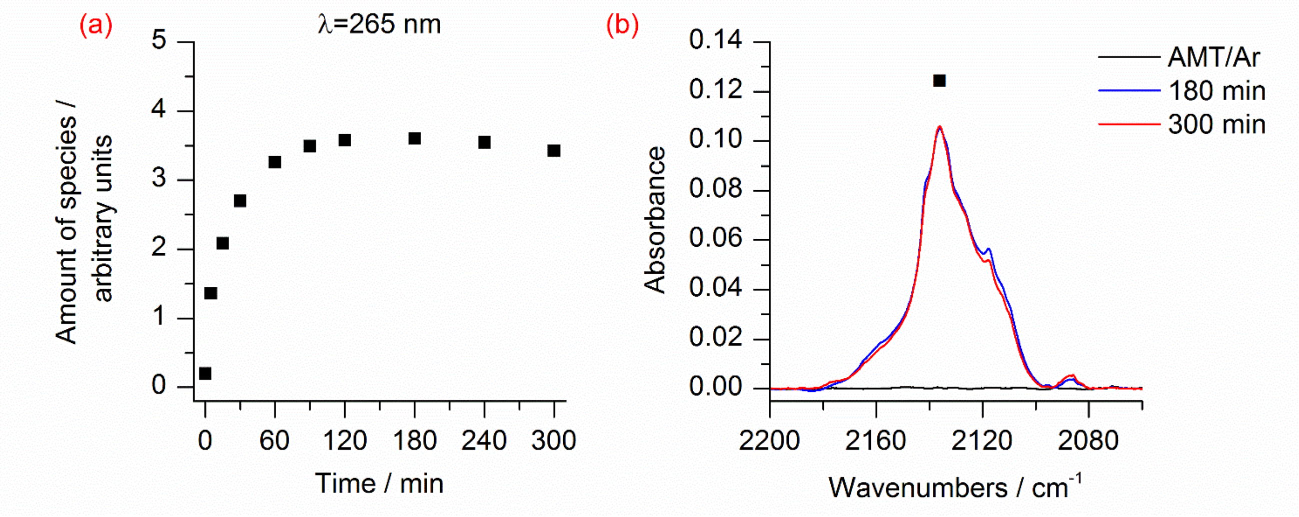

Figure S8. The 2135 cm^{-1} band in the AMT/Ar matrix spectra during irradiation at 265 nm.
